# Prevalence of potentially inappropriate prescribing in older adults in Central and Eastern Europe: a systematic review and synthesis without meta-analysis

**DOI:** 10.1038/s41598-022-19860-8

**Published:** 2022-10-06

**Authors:** Jovana Brkic, Daniela Fialova, Betul Okuyan, Ingrid Kummer, Sofija Sesto, Andreas Capiau, Maja Ortner Hadziabdic, Konstantin Tachkov, Veera Bobrova

**Affiliations:** 1 Department of Social and Clinical Pharmacy, Faculty of Pharmacy in Hradec Kralove, Charles University, Hradec Kralove, 500 05 Czech Republic; 2 Department of Geriatrics and Gerontology, First Faculty of Medicine, Charles University, Prague, 121 08 Czech Republic; 3 Clinical Pharmacy Department, Faculty of Pharmacy, Marmara University, Istanbul, 34668 Turkey; 4 Department of Social Pharmacy and Pharmaceutical Legislation, Faculty of Pharmacy, University of Belgrade, 11221 Belgrade, Serbia; 5 Pharmaceutical Care Unit, Faculty of Pharmaceutical Sciences, Ghent University, 9000 Ghent, Belgium; 6 Department of Pharmacy, Ghent University Hospital, 9000 Ghent, Belgium; 7 Centre for Applied Pharmacy, Faculty of Pharmacy and Biochemistry, University of Zagreb, 10000 Zagreb, Croatia; 8 Department of Organization and Economics of Pharmacy, Faculty of Pharmacy, Medical University of Sofia, Sofia, 1000 Bulgaria; 9 Institute of Pharmacy, Faculty of Medicine, University of Tartu, 50411 Tartu, Estonia

**Keywords:** Geriatrics, Public health

## Abstract

We aimed to systematically review the prevalence of potentially inappropriate prescribing (PIP) in older adults in Central and Eastern Europe (CEE) in all care settings. We searched Embase and MEDLINE (up to June 2019) and checked the reference lists of the included studies and relevant reviews. Eligible studies used validated explicit or implicit tools to assess the PIP prevalence in older adults in CEE. All study designs were considered, except case‒control studies and case series. We assessed the risk of bias using the Joanna Briggs Institute Prevalence Critical Appraisal Tool and the certainty of evidence using the GRADE approach. Meta-analysis was inappropriate due to heterogeneity in the outcome measurements. Therefore, we used the synthesis without meta-analysis approach—summarizing effect estimates method. This review included twenty-seven studies with 139,693 participants. Most studies were cross-sectional and conducted in high-income countries. The data synthesis across 26 studies revealed the PIP prevalence: the median was 34.6%, the interquartile range was 25.9–63.2%, and the range was 6.5–95.8%. The certainty of this evidence was very low due to the risk of bias, imprecision, and inconsistency. These findings show that PIP is a prevalent issue in the CEE region. Further well-designed studies conducted across countries are needed to strengthen the existing evidence and increase the generalizability of findings.

## Introduction

Potentially inappropriate prescribing (PIP) in older people is associated with increased morbidity, lower quality of life, increased use of health care services, and increased health care costs^1–3^. PIP, therefore, poses a clinical, humanistic and economic problem for older adults, their carers and health care systems. Furthermore, it is a prevalent global health issue in all settings of care that is likely to grow as the world population ages^4^. Although PIP is considered a highly prevalent problem worldwide, its prevalence varies widely due to differences in country contexts, health care settings, populations and measurement tools^5^. Additional information about the magnitude of the problem from relevant systematic reviews is presented in the Discussion section, illustrating that PIP is a global issue of major concern.

PIP encompasses the prescribing of potentially inappropriate medications (PIMs) and potential prescribing omissions (PPOs)^6^. PIM use refers to the prescribing of ineffective medications or medicines with higher risks than benefits (especially when safer therapeutic alternatives exist) and the prescribing of medications without a clinical indication or at the wrong dose, frequency or duration of treatment^7^. A PPO involves the omission of a clinically indicated medication^6^. The appropriateness of prescribing can be assessed using criterion-based (explicit) or judgment-based (implicit) tools^8^. Explicit tools are easily applied, reliable and reproducible but do not consider individual patient characteristics. On the other hand, implicit tools are time-consuming to use and have low reliability and reproducibility as they depend on clinician judgment but are person-specific and consider patient preferences^9^*.* The Beers criteria^7,10–14^ and Screening Tool of Older Person's Prescriptions (STOPP)^15,16^, which are explicit tools, and the Medication Appropriateness Index (MAI)^17^, which is an implicit tool, are among the most commonly used criteria to quantify prescribing appropriateness^18^. Furthermore, the Beers^7,10–14^ and STOPP^15,16^ criteria served as a basis for the development of most other validated tools^19^.

The clinical and economic consequences of PIP can be more devastating for older adults residing in regions and countries with fewer financial resources and worse health status, which contributes to deepening health inequalities globally. One such region is Central and Eastern Europe (CEE), which encompasses the following countries: Albania, Bosnia and Herzegovina, Bulgaria, Croatia, Czechia, Estonia, Hungary, Latvia, Lithuania, Montenegro, North Macedonia, Poland, Romania, Serbia, Slovakia, Slovenia, and the territory of Kosovo. In all countries in CEE, the healthy life expectancy (HALE) at age 60 (years) is lower than those in other European Union (EU) countries and other more developed countries, such as Australia, New Zealand, Canada, the Republic of Korea, Singapore and Japan (range 14.9–17.8 versus 18.2–20.4 years, respectively), except for the United States of America (16.4 years)^20^*.* Additionally, all countries in CEE have a lower standard of living, expressed as gross domestic product (GDP) per capita, purchasing power parity (PPP) (current international $), than the EU average, the Organisation for Economic Co-operation and Development (OECD) average and the high-income economies' average (48436.3, 48482.1 and 54602.9013, respectively)^21^. According to the World Bank country classification, all non-EU countries in CEE and Bulgaria are upper-middle-income economies. In contrast, the rest of the EU countries in CEE are high-income economies^22^.

PIP has been extensively explored over the past three decades, and a number of systematic reviews have been published on this topic. However, only a few systematic reviews have investigated the prevalence of PIP. Additionally, some of these systematic reviews have focused only on single countries (two systematic reviews by Bhagavathula et al.^23,24^), specific measurement tools (the systematic reviews by Hill-Taylor et al., Opondo et al., Praxedes et al., Thomas et al., and Storms et al.^25–29^), or specific sources of data (the systematic review by Guaraldo et al.^30^). Only three systematic reviews had similar inclusion and exclusion criteria but focused only on specific settings: community (Tommelein et al.^31^), primary care (Liew et al.^32^) and long-term care (LTC) (Morin et al.^33^). Furthermore, these reviews included only a few studies conducted in countries in CEE. Morin et al.^33^ included no studies from CEE, Liew et al.^32^ included only one study from CEE, and Tommelein et al.^31^ included five studies from CEE. Thus, whether the findings from these reviews, which focused on wealthier countries, can be generalized to the CEE region that encompasses former communist states with less developed medication safety programs is uncertain because of differences in country contexts, the availability of resources, and health systems.

We believe that carrying out an up-to-date comprehensive systematic review across a range of settings can inform policy-makers about the issue of PIP in the CEE region and subsequently reduce global health disparities and accelerate the development of medication safety measures in this region. Therefore, we aimed to systematically review the PIP prevalence in older adults in all care settings in countries in CEE.

## Methods

We conducted the review according to the registered protocol (PROSPERO: CRD42020152713; https://www.crd.york.ac.uk/prospero/display_record.php?RecordID=152713)^34^ and reported according to the Synthesis without meta-analysis (SWiM) in systematic reviews: reporting guideline^35^, and the Preferred reporting items for systematic reviews and meta-analyses (PRISMA) guidance^36–38^ (see Supplementary Tables S1–S4). At all stages of the review process, we contacted the study authors via email to obtain or confirm relevant information. We did not use automation tools in our review.

### Search strategy and selection criteria

We searched Embase (Embase.com; 14 June 2019) and MEDLINE (Ovid; 16 June 2019). Search strategies were adapted from two Cochrane systematic reviews^39,40^ and tailored to each database and specific interface (full search strategies are provided in Supplementary Tables S5 and S6). No filters or limits were used. Additionally, two authors independently checked the reference lists of the included studies and the reviews on similar topics. Duplicate records were removed using EndNote 20 and manually. We conducted a 'top-up' search in August 2022, and we listed the potentially eligible studies that were not incorporated into the review in the 'Studies awaiting classification' table*.*

We included studies that used validated explicit or implicit tools to measure the PIP prevalence in older adults aged 60 years and over (the United Nations standard)^41^ in all care settings in countries in CEE. We excluded studies focused on a single disease or condition, terminally ill patients and specific medications/classes of medications (because their results are not applicable to the older population as a whole). If a study reported that some participants were younger than 60 years, we attempted to contact the authors to obtain separate data for older adults (post hoc decision; see Differences between the protocol and review in Supplementary Table S7). All study designs were eligible except for case‒control studies and case series. Regarding interventional studies, participants could not be selected based on the presence of PIMs/PPOs, and only the PIP prevalence before the intervention was considered. Only primary studies published as full papers in peer-reviewed journals were included. We did not apply any language or date restrictions.

### Data collection and analysis

Two review authors independently screened the titles and abstracts to exclude clearly ineligible studies. The same two authors then independently screened the full texts of the remaining potentially relevant studies. All disagreements were resolved by discussion without the need for a third reviewer. The reviewers were not blinded to the names of the authors, their institutions or the journal of publication. Multiple reports of the same study were linked together. We used a software program when abstracts and/or articles required translation into English.

Two reviewers independently extracted data using a standardized data extraction form created and piloted specifically for this review. A third reviewer read all records in detail to check the collected data for accuracy and ensure that no relevant information was missed. This author also resolved all errors and inconsistencies, contacted the study authors and mediated consensus on disagreements. When necessary, we consulted a fourth reviewer. We collected data on the following: record details (authors, year, journal, funding sources, conflicts of interest, aims, conclusions), study characteristics (study design, sampling, recruitment, response rate, setting, country and location, number of study centers, study period, methods of data collection, sources of data, ethical approval, informed consent), participants (number, age, sex, inclusion/exclusion criteria, comorbidities, medication use), outcomes (measurement instrument, measurement instrument adaptation, timing of outcome measurements), and miscellaneous information (contact information, correspondence required and responses, comments from the reviewers). We presented key characteristics and findings of individual studies in a 'Characteristics of included studies' table, in which studies were grouped by setting and country.

The risk of bias was assessed using the Joanna Briggs Institute (JBI) Prevalence Critical Appraisal Tool^42^, which contains nine items: representativeness of the sample; appropriateness of recruitment; adequateness of the sample size; appropriateness of the description of the study subjects and setting; coverage bias; validity of the measurements; reliability of the measurements; appropriateness of statistical analysis; and adequateness of the response rate. Two authors independently applied the tool to each included study and resolved all disagreements by discussion without the need for a third reviewer. The overall risk of bias was judged as high if at least one domain was at high risk or if three domains were at unclear risk. Regarding nonreporting biases, two authors independently assessed study-level selective reporting by comparing the outcomes reported in the results to those previously specified in the aims and methods sections; protocols were not available for any of the included studies. We resolved disagreements by discussion.

The eligible outcome was the PIP prevalence measured by validated explicit or implicit tools. The PIP prevalence was defined as the proportion of persons with one or more PIMs and/or PPOs at a specified point or period in time. The PIP prevalence expressed as a proportion of prescriptions was only reported in the text of the review and excluded from the data synthesis and certainty of evidence rating. When multiple outcomes within a study were available for inclusion (the same outcome measured by different tools and/or at different time points), we reported all of them in the 'Results of individual studies' table but selected a median estimate for data synthesis. Missing prevalence estimates and confidence intervals were computed from the data collected from the studies.

We grouped all outcomes in a single analysis (not prespecified in the protocol). We did not restrict the synthesis to a subset of studies, and we did not prioritize the reporting of some study findings over other findings.

Meta-analysis was not appropriate because the measurement tools were too dissimilar across studies. Therefore, to provide a quantitative assessment, we used the statistical synthesis without meta-analysis approach—summarizing effect estimates method. In this method, each included study is represented by one outcome (in our study, in the case of multiple outcomes, the median estimate was used), and the median, interquartile range and range were calculated across studies. A limitation of this synthesis method is that equal weight is given to all studies, not accounting for differences in the sample sizes. We provide a visual display of the PIP prevalence distribution by box-and-whisker plots. We also present the results from the synthesis in the 'Summary of findings' table. Statistical synthesis was performed using IBM SPSS Statistics 27. In addition, to assess the medications that are most frequently involved in PIP, we extracted the three most frequent criteria of PIP from each study and provided a brief narrative summary.

We investigated heterogeneity visually using box-and-whisker plots. We explored the following potential sources of heterogeneity using study-level variables: study quality (studies at low risk of bias and with some concerns, and studies at high risk of bias; post hoc), study setting (acute, community, LTC, and outpatient (which includes both community-dwelling and LTC residents)), and study period (before 2010 and from 2010 onward; post hoc). We could not assess the influence of several prespecified potential modifiers, including age due to differences in reporting (mean, median, missing information), country due to a small number of studies, and measurement tools due to substantial diversity.

We decided post hoc to assess the quality of evidence related to the studies included in the data synthesis using the Grading of Recommendations Assessment, Development and Evaluation (GRADE) approach^43–45^ and created a 'Summary of findings' table. Two review authors independently judged the certainty of the evidence, with disagreements resolved by discussion. The GRADE approach specifies four categories of quality of evidence: high, moderate, low, and very low. We started at high quality because cross-sectional studies are the most appropriate research design to assess prevalence^42^. We considered downgrading the quality of evidence for each of the five GRADE domains (risk of bias, inconsistency, imprecision, indirectness, and publication bias) by one level or two levels in cases of severe problems. All our decisions are provided in the Discussion section and the footnotes of the 'Summary of findings' table.

We performed post hoc sensitivity analyses to investigate whether our decisions changed the results: 1) using the smallest and the largest outcomes instead of the median outcome for each study, and 2) excluding one study with a subset of eligible participants.

## Results

### Search results

**Figure 1:**
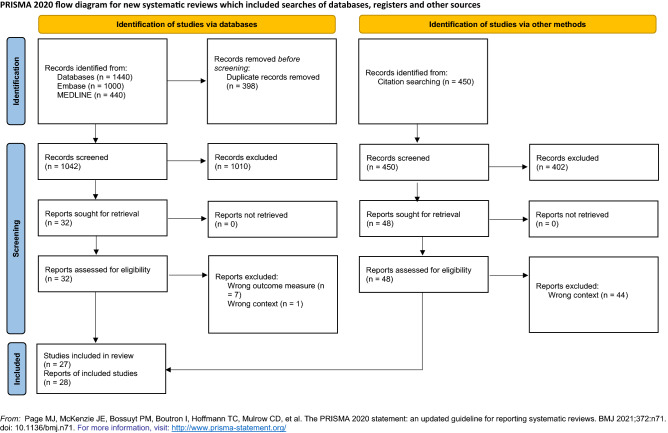
PRISMA flow diagram.

We identified 1890 records (1440 by searching electronic databases: 1000 from Embase, 440 from MEDLINE, and 450 from browsing reference lists) from our search up to 16 June 2019. After removing 398 duplicates, we screened 1492 records for eligibility and excluded 1412 records based on the title or abstract. We assessed the full text of the remaining 80 records and listed the excluded studies at this stage in the 'Characteristics of excluded studies' table (see Supplementary Table S8). Although we successfully contacted the authors of two studies, they could not provide the data on PIP prevalence (study eligibility criterion); thus, we excluded them^46,47^. Ultimately, 27 studies (28 records) met the inclusion criteria. One article reported two studies^48^. On the other hand, three papers described one study^49–51^. See the PRISMA flow diagram (shown in Fig. 1).

Our 'top-up' search yielded 637 records, of which 477 remained after duplicates were removed. Eight of the 477 screened records were potentially eligible and are listed in the 'Studies awaiting classification' table (see Supplementary Table S9).

### Characteristics of included studies

Study characteristics are summarized in the 'Characteristics of included studies' table (see Table 1). All studies were cross-sectional, except that of Stuhec et al.^52^, which was an uncontrolled before-after study. Different care settings were equally represented across studies—acute^49–51,53–58^, community^48,59–64^ and outpatient^52,65–70^ settings in seven studies, and LTC setting in six studies^48,71–75^. Only three studies were conducted in upper-middle-income countries: Serbia (2)^62,75^ and Albania (1)^54^. The rest of the studies were conducted in high-income countries: Croatia (5)^56–58,68,70^, Slovenia (5)^52,60,66,67,74^, Czechia (4)^53,59,69,72^, Poland (3)^61,63,64^, Slovakia (3)^49–51,55,73^, Romania (2)^48^, Hungary (1)^71^ and Lithuania (1)^65^. There were no studies from Bosnia and Herzegovina, Bulgaria, Estonia, Latvia, Montenegro, North Macedonia or the territory of Kosovo. Two studies were conducted internationally^53,59^, but only the data from a country in CEE were included in the review. Twelve studies were conducted up to 2010^49–51,53,55,58,59,61,63,64,67,69,70,74^, and fifteen were conducted from 2010 onward^48,52,54,56,57,60,62,65,66,68,71–73,75^.

**Table 1:** Characteristics of included studies.

Study	Country	Study design	Inclusion/exclusion criteria	Data collection	Study period (year)	Medicine category	Sample size	Female (%)	Age (years)	Number of medications	Prevalence of polypharmacy (%) (cut-off)
**Acute care setting**											
Hudhra^54^	Albania Non-EU, UMIC	Cross-sectional	60 + years & discharged from internal medicine and cardiology department	Medical charts	2013	Rx	319	43.6	M 69.7 SD 6.1 Ra 60–89	M 7.8 SD 2.2	73.0 (7 +)
Matanovic^56^	Croatia EU, HIC	Cross-sectional	65 + years & emergency admission to the internal medicine department	Medical charts & interview (patient, GP)^a^	2009–2010	Rx, OTC^b^	454	57.7	M 74.8 SD 4.2 Ra 65–94^a^	M 5.3 SD 2.9	57.5 (5 +)
Mucalo^57^	Croatia EU, HIC	Cross-sectional	65 + years & 1 + medication & hospitalised at the internal medicine department & patient/proxy capable of giving consent and communicating well	Medical charts & interview (patient/caregiver, GP); standardized data collection form	2014–2016	Rx, OTC^b^	276	49.3	M 73.9 SD 6.2 Ra 65–92^a^	M 7.8 SD 3.2^a^	91.7 (5 +)
Radosevic^58^	Croatia EU, HIC	Cross-sectional	65 + years & 1 + medication & hospitalised at the internal medicine department	Medical charts	2007^a^	Rx	142	52.1^a^	M 75.0 SD 6.3 Ra 65–97^a^	M 6.3 SD 2.7^a^	73.2 (5 +)^a^
Gallagher^53^	Czechia EU, HIC	Cross-sectional	65 + years & emergency admission to the geriatric department	Medical charts & patient assessment^a^	2008	Rx	150	65.3^c^	Mdn 82 IQR 77–86	Mdn 6 IQR 4–8	52.7 (6 +)^c^
Kostkova^55^	Slovakia EU, HIC	Cross-sectional	65 + years & hospitalised at the geriatric department & complete medical documentation; excluded who died	Medical charts	2008–2009	Rx^a^	566	62.2	M 77.4 SD 6.8	NR	68.7 admission 87.8 discharge (6 +)
Wawruch^51^* Wawruch^49^ Wawruch^50^	Slovakia EU, HIC	Cross-sectional	65 + years & hospitalised at the internal medicine department & complete medical documentation; excluded who died	Medical charts	2003–2005	Rx, OTC^a^	600	58.5	M 76.6 SD 6.5	NR	60.3 admission 62.3 discharge (6 +)
**Community setting**											
Fialova^59^	Czechia EU, HIC	Cross-sectional	65 + years & home care recipient	Patient assessment & interview (patient, caregiver) & medical charts; standardized data collection form^d^	2001–2002	Rx, OTC	428	79.0	M 81.6 SD 7.0 Ra 65–98^a^	M 6.7 SD 2.5^a^	68.5 (6 +)
Kosinska^61^	Poland EU, HIC	Cross-sectional	65 + years & 1 + medication	Prescriptions	2004	Rx	5086 (prescriptions)	64.8	M 74.5 Ra 65–100	NR	NR
Rajska-Neumann^63^	Poland EU, HIC	Cross-sectional	65 + years	Questionnaire	2002–2003^a^	Rx, OTC	1000; two cities I: 680 II: 320	65.4^c^	I: M 72.6 SD 6.5; II: M 72.5 SD 6.0	I: M 6.9 SD 3.2; II: M 6.6 SD 3.1	50.4 (7 +)
Rajska-Neumann^64^	Poland EU, HIC	Cross-sectional	100 + years	Questionnaire	1999–2000^e^	Rx, OTC	92	83.7^c^	M 101.7 SD 1.2 Ra 100–111	M 2.5 SD 2.5	32.6 (5 +)
Primejdie^48^°	Romania EU, HIC	Cross-sectional	65 + years	Prescriptions	2013	Rx	345	61.2	M 74.8 SD 6.2 Ra 65–92	Mdn 3	NR
Kovacevic^62^	Serbia Non-EU, UMIC	Cross-sectional	65 + years & 1 + medication; excluded who did not claim prescriptions personally	Interview (patient) & medical charts; standardized data collection form	2012	Rx	509	57.4	M 74.8 SD 6.5 Ra 65–95	M 5.1 SD 2.2	37.0 (6 +)
Gorup^60^	Slovenia EU, HIC	Cross-sectional	65 + years & 1 + medication & capable of giving consent and communicating well & life expectancy > 1 year	Questionnaire (patient) & patient assessment	2014–2015	Rx	503	56.7	M 74.9 SD 6.0 Ra 65–99	M 5.6 SD 2.9	62.2 (5 +)
**LTC setting**											
Kalafutova^72^	Czechia EU, HIC	Cross-sectional	65 + years & 2 + medications & capable of giving consent and communicating well	Medical charts	2012	Rx, OTC^b^	58	74.1^c^	M 82.4 SD 8.3	Rx: M 8.9 OTC: M 1.2	Rx:82.8 (6 +)^c^
Bor^71^	Hungary EU, HIC	Cross-sectional	Residing 12 + months in the LTC; excluded who died^f^	Medical charts	2010–2015	Rx, OTC^a^	184^a^	78.8^a^	M 82.6 SD 7.2 Ra 65–104^a^	M 8.5 SD 3.8^a^	91.3 (4 +)
Primejdie^48^	Romania EU, HIC	Cross-sectional	65 + years	Medical charts	2013	Rx, OTC	91	58.2	M 80.8 SD 6.8 Ra 65–98	Mdn 8	NR
Stojanovic^75^	Serbia Non-EU, UMIC	Cross-sectional	65 + years & 1 + medication	Medical charts	2018	Rx	400	69.0^c^	Mdn 83 IQR 11 Ra 65–99	Mdn 8 IQR 5	NR
Kolar^73^	Slovakia EU, HIC	Cross-sectional	65 + years	Medical charts	2014	Rx	70	58.6	M 79.9 SD 5.6 Ra 70–94^a^	M 8.1 SD 9.8^a^	90.0 (5 +)^a^
Ster^74^	Slovenia EU, HIC	Cross-sectional	65 + years & patient/proxy capable of giving consent & complete medical documentation	Medical charts; standardized data collection form	2006	Rx, OTC	2040	78.3	M 82.0 SD 7.7	M 5.8 SD 3.0	50.6 (6 +)
**Outpatient setting**											
Popovic^68^	Croatia EU, HIC	Cross-sectional	65 + years & 5 + medications	Claims database	2010	Rx	29,418	63.2	M 77 SD 5.9 Ra 65–103^a^	M 7.6 SD 1.8^a^	NA (5 + inclusion criteria)
Vlahovic-Palcevski^70^	Croatia EU, HIC	Cross-sectional	70 + years & 1 + medication	Pharmacy database	2002	Rx	10,426^a^	NR	NR	M 7.5	NR
Vinsova^69^	Czechia EU, HIC	Cross-sectional	65 + years & 1 + medication	Claims database	1997–2001	Rx	15,516	NR	NR	NR	NR
Grina^65^	Lithuania EU, HIC	Cross-sectional	65 + years & 1 + medication	Claims database	2015	Rx	431,625	68.1	M 75.8 SD 0.0	M 4.7 SD 0.0	57.5 (4 +)^g^
Jazbar^66^	Slovenia EU, HIC	Cross-sectional	65 + years & 1 + medication	Claims database	2013	Rx	345,400	60.0	M 75.4 SD 7.3 Ra 65–108^a^	M 7.8 SD 4.9^a^	72.1 (5 +)^a^
Nerat^67^	Slovenia EU, HIC	Cross-sectional	65 + years & 1 + medication	Claims database	2006	Rx	65 + : 298,990; 75 + : 136,076	NR	NR	65 + : M 7.7 SD 4.9; 75 + : M 8.3 SD 5.0	NR
Stuhec^52^	Slovenia EU, HIC	Uncontrolled before-after	65 + years & 10 + medications & medication review & complete medical documentation	Medical charts & medication review documentation	2012–2014	Rx	91	61.5	M 77.5 Mdn 78 Ra 65–91	M 13.8 Mdn 13	NA (10 + inclusion criteria)

*EU* European Union, *GP* General practitioner, *HIC* High-income country, *IQR* Interquartile range, *LTC* Long-term care, *M* Mean, *Mdn* Median, *NR *Not reported, *OTC* Over-the-counter medication, *Ra* Range, *Rx *Prescription medication, *Sd* Standard deviation, *UMIC* Upper-middle-income country.

*Indicates the major publication for the study (the study was described in three reports).

°One report described two studies.

^a^Data were obtained and/or confirmed from study authors.

^b^Dietary supplements were also included.

^c^Calculated.

^d^InterRAI Minimum Data Set for Home Care instrument, MDS-HC^76^.

^e^Data were obtained from publication by Sikora et al*.*^77^.

^f^Data for persons aged 65 + years were obtained by correspondence.

^g^Discrepancies in publication resolved in correspondence.

The 26 studies included 1,139,693 participants, ranging from 58 to 431,625 participants. One study provided results only on prescriptions (5086)^61^, and one study applied a part of the tool to patients and the other part to prescriptions (1,315,624)^68^. In studies that reported sex, the majority of participants were female (range 52.1–83.7%)^48–53,55,56,58–66,68,71–75^ except in two studies (43.6 and 49.3%)^54,57^. One study each included participants aged over 60^54^, 70^70^, and 100 years^64^, and the remaining studies included participants aged over 65 years. One study included participants aged over 50 years, but the authors provided separate data for participants aged 65 years and older^71^, which enabled us to include this study in the review.

Data were collected from different sources/combinations of sources—medical records, claim databases, pharmacy databases, prescriptions, medication review documentation, interviews, questionnaires, and patient assessments. The use of a standardized data collection form was reported in only four studies^57,59,62,74^. In most studies, only prescription medications were considered^48,52–55,58,60–62,65–70,73,75^.

Polypharmacy, or the use of multiple medications, was defined differently across the studies: in most studies, it was defined as more than four or five medicines^49–51,53,55–60,62,64,66,72–74^ and in a few studies, it was defined as more than three ^65,71^ or six medicines ^54,63^. The polypharmacy prevalence ranged from 32.6 to 91.7%^49–51,53–60,62–66,71–74^. It was not reported in seven studies^48,61,67,69,70,75^, and only adults with polypharmacy were included in two studies^52,68^.

Overall, the prevalence of PIP was reported 52 times in 27 studies, with between one and four outcomes per study. There were differences in the concepts measured (PIMs, PPOs, and both), the measurement tools used (different domains; different versions; different adaptations; combinations), and the measurement time points (admission, discharge, admission/discharge). The predominantly measured concept was PIM use; PPOs were assessed only seven times—four times separately and three times together with PIMs. Only explicit tools were used to detect PIP, namely, the Austrian consensus panel list^78^, 1997 Beers criteria^10^, 2003 Beers criteria^11^, American Geriatrics Society (AGS) 2012 Beers criteria^12^, AGS 2015 Beers criteria^13^, Comprehensive protocol^79^, 2012 CZ expert consensus criteria^80^, EU(7)-PIM list^81^, French consensus panel list^82^, Ghent Older People's Prescriptions community Pharmacy Screening (GheOP3S) tool^83^, McLeod criteria^84^, PRISCUS list^85^, Screening Tool to Alert doctors to Right Treatment (START)^15^, START criteria version 2^16^, STOPP^15^, and STOPP criteria version 2^16^. Additionally, composite tools, i.e., combinations of two or more criteria, were used in the included studies (five times). The tools used most often were different versions of the Beers criteria (21 times; and four times as a part of the composite criteria; version 2003 was used most often, 14 times and two times as a part of the composite criteria) and different versions of the STOPP criteria (seven times and two times as a part of the composite criteria). Thirteen studies used only one tool^49–52,58,60,61,63,64,68–70,72–74^, three used only a composite tool^48,71^, and the remaining eleven used more than one tool and, in some cases, more than one version of the same tool^53–57,59,62,65–67,75^. The full versions of the tools were used in only six studies^52,53,60,62,73,75^. In the remaining studies, the tools were adapted. Certain sections of the tools or individual items were excluded, most often medications that were not available on the pharmaceutical market and criteria requiring some clinical or therapeutic information (such as diagnosis, dose, dosage, and duration of treatment).

### Risk of bias in included studies

**Figure 2:**
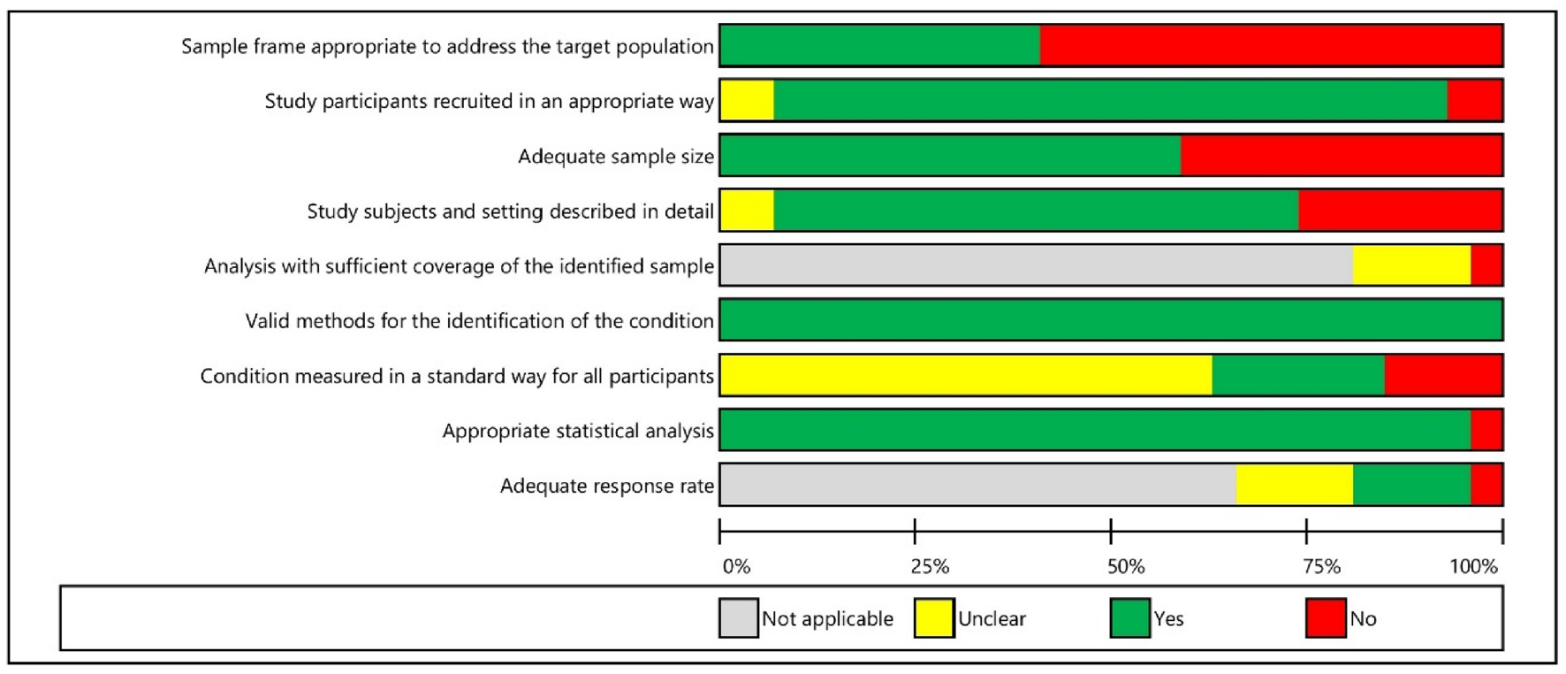
Risk of bias: reviewers' judgements about each risk of bias item across all included studies. Presented as percentages.

Only six studies were at low risk of bias or with some concerns^59,62,65,66,68,74^ (shown in Fig. 2, Supplementary Fig. S1 and Table 2). In over half of the studies^48–58,61,63,71–73,75^, the sample frame was not appropriate to address the target population (the country's older population), as it included only persons from one or several organizations. In contrast, in most studies^48–51,53–59,61,62,65–75^, participants were recruited appropriately by including everyone from the sampling frame or using random probabilistic sampling; convenience sampling was used in only a small number of studies^52,63^. The sample size was inadequate in almost half of the studies^48,52–54,57,58,64,71–73^ (determined by following the JBI Prevalence Critical Appraisal Tool recommendations). Approximately two-thirds of the studies^48–57,59,62,65,66,68,71–74^ described the study sample and setting in sufficient detail. All studies^48–75^ used valid methods (i.e., validated instruments) to assess the outcomes because this was part of the inclusion criteria. In most studies^49–51,55,56,58,63–75^, it was not clear if the condition was measured in the same, standard, reliable way for all participants. The statistical analysis, i.e., prevalence reporting, was appropriate in almost all studies^48–51,53–75^. Most studies^48–51,53–56,58,61,65–71,73,75^ used claims databases and medical records of all patients, and therefore the response rate and coverage bias assessment were not applicable to them. Furthermore, a small number of studies^59,62,72,74^ with an adequate response rate (80% or higher) had an unclear risk of coverage bias.

### Nonreporting bias

Reported outcomes were consistent with the stated aims and methods in all studies, except for the study of Kosinska et al.^61^. In this study, an additional measurement tool^10^ was mentioned in the abstract, but the outcome value was not clearly reported. We attempted to contact the study authors to clarify this, without success.

### Prevalence findings

**Figure 3:**
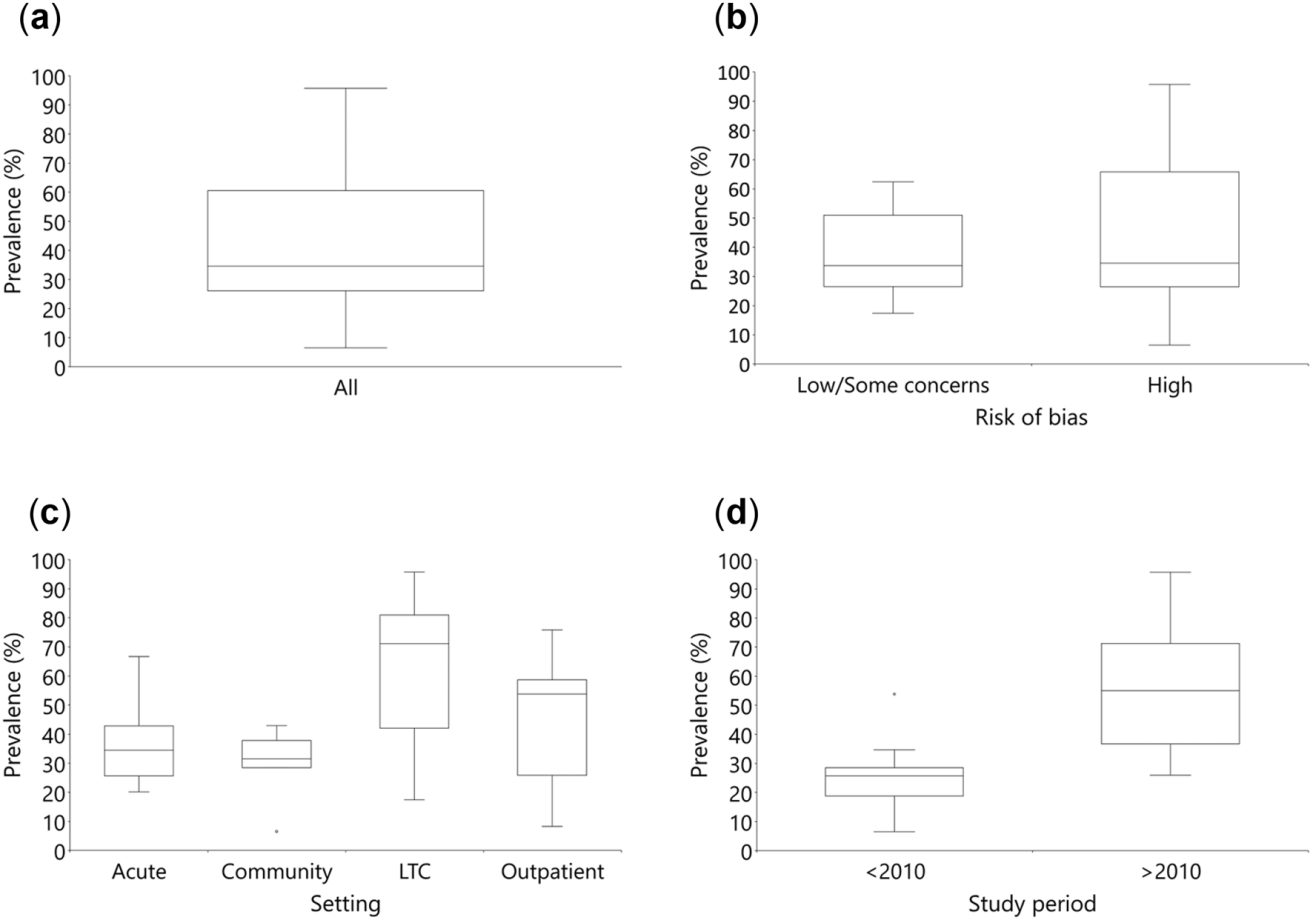
Box-and-whisker plots of prevalence of potentially inappropriate prescribing (**a**) for all outcomes, (**b**) separately by the overall risk of bias, (**c**) separately by the setting, (**d**) separately by the study period. *LTC* Long-term care.

The results of individual studies that measured the PIP prevalence in patients are presented in Table 2. The two studies that measured the PIP prevalence for prescriptions instead of patients (whose results were not used in data synthesis) reported prevalence rates of 7.4%^61^ and 2.0%^68^. The results of the data synthesis, in which 26 studies were included, showed that the median PIP prevalence in older adults residing in the CEE region was 34.6% (minimum 6.5%, maximum 95.8%, interquartile range 25.9–63.2%; 1,139,693 participants; very low certainty of evidence)^48–60,62–75^ (see Table 3 and Fig. 3a).

**Table 2:** Results of individual studies.

Study	Country	Risk of bias	Tool	Tool adaptation	Number of patients with 1 + PIP	Sample size	Prevalence of patients with 1 + PIP (95% CI)	Timing of outcome measurement
**Acute care setting**								
Hudhra^54^	Albania	H	Beers 2012^12^	Independent and considering diagnosis & registered & excluding criteria requiring follow-up data^a^	110	319	0.34 (0.29–0.40)^b^	Discharge
			STOPP^15^		110	319	0.34 (0.29–0.40)^b^	Discharge
			STOPP version 2^16^		201	319	0.63 (0.58–0.68)^b^	Discharge
Matanovic^56^	Croatia	H	Beers 2012^12^	Independent and considering diagnosis	263	454	0.58 (0.53–0.62)^b^	Admission^a^
			Comprehensive protocol^79^		200	454	0.44 (0.40–0.49)^b^	Admission^a^
Mucalo^57^	Croatia	H	Comprehensive protocol^79^	Independent and considering diagnosis & registered	102	276	0.37 (0.31–0.43)^b^	Discharge
			EU(7)-PIM list^81^		184	276	0.67 (0.61–0.72)^b^	Discharge
			STOPP version 2 ^16^		190	276	0.69 (0.63–0.74)^b^	Discharge
Radosevic^58^	Croatia	H	Beers 2003^11^	Independent of diagnosis & registered	35	142	0.25 (0.18–0.32)^b^	During hospitalisation
Gallagher^53^	Czechia	H	Beers 2003^11^	All criteria	34	150	0.23 (0.17–0.30)^b^	Admission
			START^15^		81	150	0.54 (0.46–0.62)^b^	Admission
			STOPP^15^		52	150	0.35 (0.27–0.43)^b^	Admission
Kostkova^55^	Slovakia	H	Beers 2003^11^	Independent of diagnosis	128	566	0.23 (0.19–0.26)^b^	Admission
					157	566	0.28 (0.24–0.32)^b^	Discharge
			French list^82^		145	566	0.26 (0.22–0.29)^b^	Admission
					172	566	0.30 (0.27–0.34)^b^	Discharge
Wawruch^51^ *	Slovakia	H	Beers 2003^11^	Independent of diagnosis^a^	121	600	0.20 (0.17–0.24)^b^	Admission
					120	600	0.20 (0.17–0.23)^b^	Discharge
					126	600	0.21 (0.18–0.24)^b^	Admission & discharge
**Community setting**								
Fialova^59^	Czechia	L	Beers 1997^10^	Independent of diagnosis & registered & excluding criteria concerning DDIs and requiring duration of use	67	428	0.16 (0.13–0.19)^b^	N/A
			Beers 2003^11^		108	428	0.25 (0.21–0.30)^b^	N/A
			McLeod^84^		136	428	0.32 (0.28–0.36)^b^	N/A
			Composite: Beers 1997^10^& Beers 2003^11^ & McLeod^84^		176	428	0.41 (0.37–0.46)^b^	N/A
Rajska-Neumann^63^	Poland	H	Beers 1997^10^	Independent and considering diagnosis & excluding criteria requiring dose, dosage, duration of use^a^	285	1000	0.28 (0.26–0.31)^b,c^	N/A
Rajska-Neumann^64^	Poland	H	Beers 2003^11^	Independent of diagnosis	6	92	0.07 (0.03–0.14)^b^	N/A
Primejdie^48^°	Romania	H	Composite: PRISCUS list^85^ & START^15^ & STOPP^15^	Registered & excluding criteria requiring clinical information and concerning OTCs	119	345	0.34 (0.30–0.40)^b^	N/A
Kovacevic^62^	Serbia	L	START^15^	All criteria	257	509	0.50 (0.46–0.55)^b^	N/A
			STOPP ^15^		139	509	0.27 (0.24–0.31)^b^	N/A
Gorup^60^	Slovenia	H	START^15^	All criteria	216	503	0.43 (0.39–0.47)^b^	N/A
**LTC setting**								
Kalafutova^72^	Czechia	H	STOPP^15^	NR	38	58	0.66 (0.53–0.77)^b^	N/A
Bor^71^	Hungary	H	Composite: Austrian list^78^ & Beers 2015^13^ & French list^82^ & PRISCUS list^85^	Independent of diagnosis & registered^a^	141	184	0.77 (0.70–0.82)^b,d^	N/A
Primejdie^48^°	Romania	H	Composite: PRISCUS list^85^ & START^15^ & STOPP^15^	Registered & excluding criteria requiring clinical information	75	91	0.82 (0.73–0.89)^b^	N/A
Stojanovic^75^	Serbia	H	GheOP3S tool^83^	All criteria	383	400	0.96 (0.93–0.97)^b^	N/A
			START version 2^16^		399	400	1.00 (0.98–1.00)^b^	N/A
			STOPP version 2^16^		344	400	0.86 (0.82–0.89)^b^	N/A
Kolar^73^	Slovakia	H	2012 CZ criteria^80^	All criteria^a^	24	70	0.34 (0.24–0.46)^b^	N/A
Ster^74^	Slovenia	SC	Beers 2003^11^	Independent and considering diagnosis & high severity rating & registered	355	2040	0.17 (0.16–0.19)^b^	N/A
**Outpatient setting**								
Popovic^68^	Croatia	SC	Comprehensive protocol^79^	Independent of diagnosis	18,358	29,418	0.62 (0.62–0.63)^b,e^	N/A
Vlahovic-Palcevski^70^	Croatia	H	Beers 1997^10^	Independent of diagnosis & registered & excluding criteria requiring dosage, duration of use	864	10,426	0.08 (0.08–0.09)^a,b^	N/A
Vinsova^69^	Czechia	H	Beers 2003^11^	Independent of diagnosis & registered & excluding criteria requiring dose, dosage, duration of use	8351	15,516	0.54 (0.53–0.55)^b^	N/A
Grina^65^	Lithuania	SC	Beers 2003^11^	Independent of diagnosis & registered, reimbursed & excluding criteria concerning DDIs and requiring clinical information	111,859	431,625	0.26 (0.26–0.26)^b^	N/A
			Beers 2015^13^		104,126	431,625	0.24 (0.24–0.24)^b^	N/A
			EU(7)-PIM list^81^		246,724	431,625	0.57 (0.57–0.57)^b,c^	N/A
Jazbar^66^	Slovenia	SC	Austrian list^78^	Independent of diagnosis & excluding criteria requiring dose	187,186	345,400	0.54 (0.54–0.54)^b^	N/A
			Beers 2012^12^		192,588	345,400	0.56 (0.56–0.56)^b^	N/A
			EU(7)-PIM list^81^		208,085	345,400	0.60 (0.60–0.60)^b^	N/A
			PRISCUS list^85^		122,255	345,400	0.35 (0.35–0.36)^b^	N/A
Nerat^67^	Slovenia	H	Beers 2003^11^	Independent of diagnosis	66,994	298,990	0.22 (0.22–0.23)^b^	N/A
			French list^82^		34,999	136,076	0.26 (0.25–0.26)^b^	N/A
			Composite: Beers 2003^11^ & French list^82^		48,917	136,076	0.36 (0.36–0.36)^b^	N/A
Stuhec^52^	Slovenia	H	PRISCUS list^85^	All criteria^a^	69	91	0.76 (0.66–0.84)^b, f^	N/A

*DDI* Drug-drug interaction, *GheOP3S* Ghent Older People's Prescriptions community Pharmacy Screening, *H* High, *L* Low, *LTC* Long-term care, *N/A* Not applicable, *SC* Some concerns, *START* Screening Tool to Alert doctors to Right Treatment, *STOPP* Screening Tool of Older Person's Prescriptions.

*Indicates the major publication for the study (the study was described in three reports).

°One report described two studies.

^a^Data were obtained and/or confirmed from study authors.

^b^Calculated.

^c^Discrepancies in publication resolved in correspondence.

^d^Data for persons aged 65 + years were obtained by correspondence.

^e^A part of the criteria considering diagnosis was presented separately as a percentage of a total number of prescriptions (1,315,624) that was 2.0%.

^f^Data were obtained from the master's thesis by Gorenc^86^.

**Table 3:** Summary of findings.

Outcomes	Median (range)	Number of participants (studies)	Certainty of the evidence (GRADE)	Comments
The proportion of patients with one or more potentially inappropriate medications (PIMs) and/or potential prescribing omissions (PPOs) Assessed with: explicit validated tools	34.6% (6.5–95.8)^a^	1,139,693 (26)	⊕ ⊝ ⊝ ⊝ VERY LOW^b^	Austrian consensus panel list^78^, 1997 Beers criteria^10^, 2003 Beers criteria^11^, American Geriatrics Society (AGS) 2012 Beers criteria^12^, AGS 2015 Beers criteria^13^, Comprehensive protocol^79^, 2012 CZ expert consensus criteria^80^, EU(7)-PIM list^81^, French consensus panel list^82^, Ghent Older People's Prescriptions community Pharmacy Screening (GheOP3S) tool^83^, McLeod criteria^84^, PRISCUS list^85^, Screening Tool to Alert doctors to Right Treatment (START)^15^, START criteria version 2^16^, Screening Tool of Older Person's Prescriptions (STOPP)^15^, STOPP criteria version 2^16^ and composite tools (combinations of two or more tools)

GRADE Working Group grades of evidence: High quality – we are very confident that the true effect lies close to that of the estimate of the effect; Moderate quality – we are moderately confident in the effect estimate: the true effect is likely to be close to the estimate of the effect, but there is a possibility that it is substantially different; Low quality – our confidence in the effect estimate is limited: the true effect may be substantially different from the estimate of the effect; Very low quality – we have very little confidence in the effect estimate: the true effect is likely to be substantially different from the estimate of effect.

^a^One study by Kosinska et al.^61^ and part of the results from one study by Popovic et al.^68^ were excluded from the analysis because a unit of analysis was prescription, not a patient.

^b^We downgraded the evidence three levels from high to very low due to the risk of bias (most studies at high or unclear risk of bias), imprecision (number of studies with small sample sizes), and inconsistency (considerable heterogeneity).

Benzodiazepines were among the top three most frequently used PIMs among almost all studies and all tools. The omission of statins for primary prevention in diabetes mellitus was among the top three PPOs in all studies^48,53,60,62^ using the START criteria version 1^15^. However, this item was removed from the revised START criteria version 2^16^ due to the lack of evidence. Only one study (Stojanovic et al.^75^) used other tools to assess PPOs, the START criteria version 2^16^ and the GheOP3S tool^83^, which both detected a lack of vaccination as the biggest issue.

### Heterogeneity assessment

An informal visual examination of heterogeneity suggested that the PIP prevalence is similar between studies at high risk of bias (20 studies)^48–58,60,63,64,67,69–73,75^ and those at low risk of bias or with some concerns (six studies)^59,62,65,66,68,74^. Furthermore, visual examination of the box-and-whisker plots showed that the PIP prevalence may be higher in LTC (six studies)^48,71–75^ and outpatient settings (seven studies)^52,65–70^ than in acute (seven studies)^49–51,53–58^ and community care settings (six studies)^48,59,60,62–64^. Finally, when informally exploring heterogeneity, we found that the prevalence might be higher in studies from 2010 onward (15 studies)^48,52,54,56,57,60,62,65,66,68,71–73,75^ than before 2010 (11 studies)^49–51,53,55,58,59,63,64,67,69,70,74^ (see Fig. 3).

### Sensitivity analyses

The PIP prevalence remained consistent with the primary analysis when we reanalyzed the data using the smallest outcome from each study. However, when we used the largest outcome from each study, the PIP prevalence increased. Furthermore, the PIP prevalence remained almost unchanged when we excluded the study with a subset of relevant participants^71^ (see Supplementary Table S10).

## Discussion

This systematic review is the first to estimate the PIP prevalence in older adults across all settings and medications in one region, countries in CEE. We identified that the issue of PIP in older adults was not comprehensively studied in the CEE region, particularly in upper-middle-income countries. Among twenty-six studies^48–60,62–75^, the median prevalence of PIP in older adults in the CEE region was 34.6% (interquartile range 25.9–63.2%, 26 studies, 139,693 participants, very low certainty of evidence), determined by data synthesis using the summarizing effect estimates method. Thus, our findings suggest that PIP in older adults is a highly prevalent problem and our informal visual examination of heterogeneity showed that the prevalence of PIP was higher in LTC^48,71–75^ and outpatient settings^52,65–70^ than in acute^49–51,53–58^ and community care settings^48,59,60,62–64^.

Our results are in agreement with those obtained in reviews that used similar inclusion/exclusion criteria and showed PIP prevalences of 22.6% in community-dwelling older persons from Europe^31^, 33.3% in older persons in primary care settings worldwide^32^, and 43.2% in older persons residing in LTC settings worldwide^33^. The results of the review by Morin et al. showed that the prevalence of PIP in LTC residents varied across regions: 49.0% in Europe, 26.8% in North America and 29.8% in other countries^33^. The variance of the PIP prevalence across countries was also described in the review by Liew et al.: the United Kingdom, Belgium, Australia, and New Zealand had higher PIP prevalences (35.9–59.2%) than the United States, Canada, the Netherlands, and middle-income countries (23.2–29.9%)^32^. Furthermore, we observed a large variation in the prevalence of PIP across studies (from 6.5 to 95.8%), which is consistent with previous reviews; in the review by Morin et al., the PIP prevalence ranged from 5.4 to 95%^33^, and in the review by Tommelein et al., it ranged from 0.0 to 98.8%^31^. We found that benzodiazepines were the most frequently prescribed PIMs, which is in agreement with the findings of Morin et al.^33^ and Tommelein et al.^31^. Only the systematic review by Tommelein et al.^31^ discussed the most prevalent PPOs and found, as we did, that the omission of statins for primary prevention in diabetes mellitus was the most prevalent. The systematic review by Liew et al.^32^ did not report which medications were most frequently involved in PIP.

Our systematic review is more comprehensive than the above-stated reviews^31–33^ regarding the CEE region because we included 22 studies from CEE that were not reported in these reviews. However, we excluded a study by Primejdie et al.^46^ reported in the review by Tommelein et al.^31^ because the author could not provide complete outcome data. Furthermore, our review differs from these reviews in several important methodological aspects: (1) the multiplicity of outcomes: when multiple outcomes per study were available, Tommelein et al.^31^ and Morin et al.^33^ did not select one outcome or use a statistical method that accounted for the dependency; on the other hand, Liew et al.^32^ used multilevel modeling to address the dependency among multiple prevalence estimates from each study; (2) data synthesis: they pooled data using a random-effect method, which we considered inappropriate in our review; (3) risk of bias: they assessed risk of bias with an adapted version of the Strengthening the Reporting of Observational Studies in Epidemiology (STROBE) Statement: guidelines for reporting observational studies^87^ (Morin et al.^33^), 'a slightly adapted quality assessment scale from the Cochrane Collaboration group' (Tommelein et al.^31^) and the Newcastle‒Ottawa Scale (NOS) for assessing the quality of nonrandomised studies in meta-analyses ^88^ (Liew et al.^32^); and (4) certainty of the evidence: they did not rate the certainty of evidence (although Liew et al.^32^ stated in their protocol^89^ that they would use the GRADE approach^43–45^). However, despite the differences between our review and these reviews, we agree with their conclusions that the PIP prevalence in older adults is high. Additionally, our systematic review showed an increasing trend of PIP over the years, which is in line with the findings obtained by Liew et al.^32^ and Morin et al.^33^. We agree with these authors that the increasing prevalence of PIP over time might be due to the increased comprehensiveness of measurement tools, which are able to identify more prescribing problems.

Our review supports findings from the other two systematic reviews^31,33^ that the long-term use of benzodiazepines and the use of long-acting benzodiazepines are still highly prevalent among older adults. Benzodiazepine use in older adults is associated with cognitive impairment, sedation, delirium, dependence, withdrawal syndrome, and psychomotor impairment that increases the risk of motor vehicle accidents and falls^90,91^. Two especially important negative outcomes of benzodiazepine use in older adults are falls and fall-related fractures because they are common and important causes of morbidity, mortality, hospitalization, and admission to LTC facilities. Therefore, greater and continued efforts are needed to rationalize benzodiazepine prescribing.

PIP prevalence estimates vary widely across studies for several reasons. The included studies were heterogeneous regarding the inclusion criteria, participants and contexts. Regarding health status, participants varied across studies due to the different inclusion and exclusion criteria that were applied: some studies included higher-risk individuals (e.g., persons with polypharmacy), and some included healthier individuals (e.g., without cognitive impairment). Additionally, countries in CEE differ in the following aspects, which might have changed over time: health and social care systems; legislation and regulations; pharmaceutical pricing and reimbursement models; prescribing practices; the availability of medications considered PIMs in pharmaceutical markets; the availability of medication safety policies, strategies and practices; the availability of medication review and deprescribing services; the availability of interdisciplinary care models; and the availability of health care professionals who are educated and trained in various aspects of geriatrics and geriatric pharmacotherapy. We also noted variation in the types of medication regimens to which the instruments were applied, with most studies using only prescription medicines, which may also impact the PIP prevalence. However, the most important difference between the studies was the considerable variation in outcome measurements, which precluded meta-analysis. Thus, we suggest using validated measurement tools with all their items, which would enable more meaningful comparisons between studies and meta-analyses.

Studies on PIP in older patients residing in the CEE region were conducted across care settings, increasing the generalizability of our findings. However, our findings may be more applicable to high-income countries in CEE because we identified only three studies from upper-middle-income countries. None of the included studies used implicit tools to measure the PIP prevalence. Thus, the results of this review are not applicable to this type of outcome measurement.

We downgraded the certainty of evidence from high to very low for several reasons. First, most studies were at high or unclear risk of bias in one or more risk of bias domains; thus, we downgraded the quality of evidence by one level. Second, although the appropriateness of the sample size was part of the risk of bias assessment, we decided to downgrade the quality of evidence by one level for imprecision. Third, variation in the prevalence estimates across studies was considerable, and consequently, we downgraded the quality of evidence for inconsistency by one level. Finally, we did not downgrade the quality of evidence for the following: minor issues with indirectness (most studies were from high-income countries, and only explicit tools were used) and the possibility of publication bias.

The strength of this review is that we followed the methods outlined in the Cochrane Handbook for Systematic Reviews of Interventions version 6.1 (updated September 2020)^92^. Furthermore, when potential conflicts of interest existed because the review authors were involved in the studies we considered for inclusion, we excluded these authors from screening, data extraction and the risk of bias assessment.

Although we tried to limit bias at every stage of the review, some limitations remain. First, the risk of publication bias may be considerable due to our decision to include only studies published as full papers in peer-reviewed journals. We thought this would be the most reproducible and transparent approach due to a large volume of gray literature with unverified quality in this area and the absence of study registers and protocols. Second, two authors could not provide the necessary outcome data, and we excluded these studies^46,47^. Finally, another potential limitation is that we did not fully incorporate the studies from our 'top-up' search into the review.

## Conclusions

These results suggest that PIP in older adults is a prevalent problem throughout the CEE region. However, our findings must be interpreted with caution due to the very low certainty of the evidence.

Our review's findings could be used to raise awareness among policymakers, health care professionals, and the general public about the prevalent issue of PIP in older adults, which should be addressed in the near future at the national and international levels. Public health authorities should bring together all stakeholders to tackle this problem, primarily by raising awareness and educating health care professionals and the public about the problem of PIP in older adults and about the validated tools that should be used to minimize this issue and its negative consequences.

More research is needed to strengthen the existing evidence and increase the generalizability of the findings. Further studies should be of high-level quality, i.e., where applicable, the sample size should be calculated, probabilistic sampling should be used, a representative sample should be obtained, the response rate should be calculated, and the differences between responders and non-responders should be examined. Additionally, studies should be conducted in different care settings and countries, particularly in upper-middle-income countries where the evidence is scarce. Finally, studies should be clearly reported using appropriate guidelines.

## Supplementary Information

Supplementary Information.

## Data Availability

All data generated or analyzed during this study are included in this published article (and its Supplementary Information files).

## References

[1] Mekonnen AB, Redley B, de Courten B, Manias E (2021). Potentially inappropriate prescribing and its associations with health-related and system-related outcomes in hospitalised older adults: A systematic review and meta-analysis. Br. J. Clin. Pharmacol..

[2] Liew TM, Lee CS, Goh Shawn KL, Chang ZY (2019). Potentially inappropriate prescribing among older persons: A meta-analysis of observational studies. Ann. Fam. Med..

[3] Malakouti SK (2021). A systematic review of potentially inappropriate medications use and related costs among the elderly. Value Health Reg. Issues.

[4] Cullinan S, O’Mahony D, Fleming A, Byrne S (2014). A meta-synthesis of potentially inappropriate prescribing in older patients. Drugs Aging.

[5] Hill-Taylor B (2016). Effectiveness of the STOPP/START (Screening Tool of Older Persons’ potentially inappropriate Prescriptions/Screening Tool to Alert doctors to the Right Treatment) criteria: Systematic review and meta-analysis of randomized controlled studies. J. Clin. Pharm. Ther..

[6] O’Connor MN, Gallagher P, Omahony D (2012). Inappropriate prescribing: Criteria, detection and prevention. Drugs Aging.

[7] Beers MH (1991). Explicit criteria for determining inappropriate medication use in nursing home residents. Arch. Intern. Med..

[8] Spinewine A (2007). Appropriate prescribing in elderly people: How well can it be measured and optimised?. Lancet.

[9] Dimitrow MS, Airaksinen MSA, Kivelä S-L, Lyles A, Leikola SNS (2011). Comparison of prescribing criteria to evaluate the appropriateness of drug treatment in individuals aged 65 and older: A systematic review. J. Am. Geriatr. Soc..

[10] Beers MH (1997). Explicit criteria for determining potentially inappropriate medication use by the elderly an update. Arch. Intern. Med..

[11] Fick DM (2003). Updating the beers criteria for potentially inappropriate medication use in older adults: Results of a US consensus panel of experts. Arch. Intern. Med..

[12] American Geriatrics Society Beers Criteria Update Expert Panel (2012). American geriatrics society updated beers criteria for potentially inappropriate medication use in older adults. J. Am. Geriatr. Soc..

[13] American Geriatrics Society Beers Criteria Update Expert Panel (2015). American geriatrics society 2015 updated beers criteria for potentially inappropriate medication use in older adults. J. Am. Geriatr. Soc..

[14] 2019 American Geriatrics Society Beers Criteria® Update Expert Panel. American Geriatrics Society 2019 updated AGS Beers Criteria® for potentially inappropriate medication use in older adults. *J. Am. Geriatr. Soc.* **67**, 674–694 (2019). 10.1111/jgs.15767 30693946

[15] Gallagher P, Ryan C, Byrne S, Kennedy J, O’Mahony D (2008). STOPP (Screening Tool of Older Person’s Prescriptions) and START (Screening Tool to Alert doctors to Right Treatment). Consensus validation. Int. J. Clin. Pharmacol. Ther..

[16] O’Mahony D (2015). STOPP/START criteria for potentially inappropriate prescribing in older people: Version 2. Age Ageing.

[17] Hanlon JT (1992). A method for assessing drug therapy appropriateness. J. Clin. Epidemiol..

[18] Clyne B (2016). Interventions to address potentially inappropriate prescribing in community-dwelling older adults: A systematic review of randomized controlled trials. J. Am. Geriatr. Soc..

[19] Motter FR, Fritzen JS, Hilmer SN, Paniz ÉV, Paniz VMV (2018). Potentially inappropriate medication in the elderly: A systematic review of validated explicit criteria. Eur. J. Clin. Pharmacol..

[20] World Health Organization. Global Health Observatory data repository. Healthy life expectancy (HALE) Data by country. https://apps.who.int/gho/data/view.main.HALEXv?lang=en.

[21] World Bank. GDP per capita, PPP (current international $). https://data.worldbank.org/indicator/NY.GDP.PCAP.PP.CD.

[22] World Bank. World Bank Country and Lending Groups. https://datahelpdesk.worldbank.org/knowledgebase/articles/906519-world-bank-country-and-lending-groups.

[23] Bhagavathula AS (2021). Prevalence of polypharmacy, hyperpolypharmacy and potentially inappropriate medication use in older adults in India: A systematic review and meta-analysis. Front. Pharmacol..

[24] Bhagavathula AS, Gebreyohannes EA, Fialova D (2022). Prevalence of polypharmacy and risks of potentially inappropriate medication use in the older population in a developing country: A systematic review and meta-analysis. Gerontology.

[25] Hill-Taylor B (2013). Application of the STOPP/START criteria: A systematic review of the prevalence of potentially inappropriate prescribing in older adults, and evidence of clinical, humanistic and economic impact. J. Clin. Pharm. Ther..

[26] Opondo D (2012). Inappropriateness of medication prescriptions to elderly patients in the primary care setting: A systematic review. PLoS ONE.

[27] Praxedes, M. F. D. S., Pereira, G. C. D. S., Lima, C. F. D. M., Santos, D. B. D. & Berhends, J. S. Prescribing potentially inappropriate medications for the elderly according to Beers Criteria: Systematic review. * Cien. Saude Colet.* **26**, 3209–3219 (2021). 10.1590/1413-81232021268.05672020 34378710

[28] Thomas RE, Thomas BC (2019). A systematic review of studies of the STOPP/START 2015 and American Geriatric Society Beers 2015 criteria in patients≥ 65 years. Curr. Aging Sci..

[29] Storms H, Marquet K, Aertgeerts B, Claes N (2017). Prevalence of inappropriate medication use in residential long-term care facilities for the elderly: A systematic review. Eur. J. Gen. Pract..

[30] Guaraldo L, Cano FG, Damasceno GS, Rozenfeld S (2011). Inappropriate medication use among the elderly: A systematic review of administrative databases. BMC Geriatr..

[31] Tommelein E (2015). Potentially inappropriate prescribing in community-dwelling older people across Europe: A systematic literature review. Eur. J. Clin. Pharmacol..

[32] Liew TM, Lee CS, Goh SKL, Chang ZY (2020). The prevalence and impact of potentially inappropriate prescribing among older persons in primary care settings: Multilevel meta-analysis. Age Ageing.

[33] Morin L, Laroche ML, Texier G, Johnell K (2016). Prevalence of potentially inappropriate medication use in older adults living in nursing homes: A systematic review. J. Am. Med. Dir. Assoc..

[34] Brkic, J. *et al.* Prevalence of potentially inappropriate medication use in older adults in Central and Eastern Europe: a systematic review. PROSPERO 2020 CRD42020152713. https://www.crd.york.ac.uk/prospero/display_record.php?ID=CRD42020152713 (2020).

[35] Campbell M (2020). Synthesis without meta-analysis (SWiM) in systematic reviews: Reporting guideline. BMJ.

[36] Page MJ (2021). The PRISMA 2020 statement: An updated guideline for reporting systematic reviews. BMJ.

[37] Page MJ (2021). PRISMA 2020 explanation and elaboration: Updated guidance and exemplars for reporting systematic reviews. BMJ.

[38] Rethlefsen ML (2021). PRISMA-S: An extension to the PRISMA statement for reporting literature searches in systematic reviews. Syst. Rev..

[39] Alldred DP, Kennedy MC, Hughes C, Chen TF, Miller P (2016). Interventions to optimise prescribing for older people in care homes. Cochrane Database Syst. Rev..

[40] Rankin A (2018). Interventions to improve the appropriate use of polypharmacy for older people. Cochrane Database Syst. Rev..

[41] World Health Organization. *Active Ageing: A Policy Framework*. (World Health Organization, 2002). 12040973

[42] Munn Z, Moola S, Lisy K, Riitano D, Tufanaru C (2015). Methodological guidance for systematic reviews of observational epidemiological studies reporting prevalence and cumulative incidence data. Int. J. Evid. Based Healthc..

[43] Guyatt GH (2008). GRADE: An emerging consensus on rating quality of evidence and strength of recommendations. BMJ.

[44] Guyatt G (2011). GRADE guidelines: 1. Introduction—GRADE evidence profiles and summary of findings tables. J. Clin. Epidemiol..

[45] GRADE Working Group (2004). Grading quality of evidence and strength of recommendations. BMJ.

[46] Primejdie D, Bojiţǎ M, Popa A (2012). Potential inappropriate medication use in community - dwelling elderly patients. A qualitative study. Farmacia.

[47] Ilić D, Bukumirić Z, Janković S (2015). Impact of educational intervention on prescribing inappropriate medication to elderly nursing homes residents. Srp. Arh. Celok. Lek..

[48] Primejdie DP, Bojita MT, Popa A (2016). Potentially inappropriate medications in elderly ambulatory and institutionalized patients: An observational study. BMC Pharmacol. Toxicol..

[49] Wawruch M (2006). Quality indicators of pharmacotherapy in geriatrics. Klin Farmakol Farm.

[50] Wawruch M (2008). Factors influencing the use of potentially inappropriate medication in older patients in Slovakia. J. Clin. Pharm. Ther..

[51] Wawruch M (2006). Perception of potentially inappropriate medication in elderly patients by Slovak physicians. Pharmacoepidemiol. Drug Saf..

[52] Stuhec M, Gorenc K, Zelko E (2019). Evaluation of a collaborative care approach between general practitioners and clinical pharmacists in primary care community settings in elderly patients on polypharmacy in Slovenia: A cohort retrospective study reveals positive evidence for implementation. BMC Health Serv. Res..

[53] Gallagher P (2011). Prevalence of potentially inappropriate prescribing in an acutely ill population of older patients admitted to six European hospitals. Eur. J. Clin. Pharmacol..

[54] Hudhra K (2016). Prevalence and factors associated with potentially inappropriate prescriptions among older patients at hospital discharge. J. Eval. Clin. Pract..

[55] Kostková L, Mačugová A, Drobná V, Dukát A, Wawruch M (2011). Potentially inappropriate prescription in elderly patients: Comparison of selected quality indicators. Klin. Farmakol. Farm..

[56] Matanović SM, Vlahović-Palčevski V (2014). Potentially inappropriate prescribing to the elderly: Comparison of new protocol to Beers criteria with relation to hospitalizations for ADRs. Eur. J. Clin. Pharmacol..

[57] Mucalo I (2017). Potentially inappropriate medicines in elderly hospitalised patients according to the EU(7)-PIM list, STOPP version 2 criteria and comprehensive protocol. Eur. J. Clin. Pharmacol..

[58] Radošević N, Gantumur M, Vlahović-Palčevski V (2008). Potentially inappropriate prescribing to hospitalised patients. Pharmacoepidemiol. Drug Saf..

[59] Fialová D (2005). Potentially inappropriate medication use among elderly home care patients in Europe. JAMA.

[60] Gorup EC, Šter MP (2017). Number of medications or number of diseases: What influences underprescribing?. Eur. J. Clin. Pharmacol..

[61] Kosińska K, Brandys J (2007). Potentially inappropriate drugs for geriatric patients. Przegla̧d Lek..

[62] Kovačević SV (2014). Potentially inappropriate prescribing in older primary care patients. PLoS ONE.

[63] Rajska-Neumann A, Wieczorowska-Tobis K (2007). Polypharmacy and potential inappropriateness of pharmaco-logical treatment among commuinity-dwellling elderly patients. Arch. Gerontol. Geriatr..

[64] Rajska-Neumann A (2011). Drug consumption among Polish centenarians. Arch. Gerontol. Geriatr..

[65] Grina D, Briedis V (2017). The use of potentially inappropriate medications among the Lithuanian elderly according to Beers and EU(7)-PIM list – a nationwide cross-sectional study on reimbursement claims data. J. Clin. Pharm. Ther..

[66] Jazbar J, Locatelli I, Kos M (2017). Extent and nature of inappropriate medication prescribing among elderly in Slovenia. Farm. Vestn..

[67] Nerat T, Kos M (2011). Analysis of inappropriate medication prescribing in Slovenian elderly patients based on the Beers and Laroche criteria. Zdr. Varst..

[68] Popović B (2014). Potentially inappropriate prescribing in elderly outpatients in Croatia. Eur. J. Clin. Pharmacol..

[69] Vinšová J (2006). Prevalence and longitudinal trends in prescription of potentially inappropriate medications for the elderly in the Czech Republic. Prakt. Lek..

[70] Vlahović-Palčevski V, Bergman U (2004). Quality of prescribing for the elderly in Croatia - Computerized pharmacy data can be used to screen for potentially inappropriate prescribing. Eur. J. Clin. Pharmacol..

[71] Bor A (2017). Medication use and risk of falls among nursing home residents: A retrospective cohort study. Int. J. Clin. Pharm..

[72] Kalafutová S, Šulcová H, Jurašková B, Vlček J (2014). A pharmacotherapy of nursing home residents. Geriatr. Gerontol..

[73] Kolar J, Tinkova B, Ambrus T, Tinkova V (2018). Analysis of pharmacotherapy in senior homes residents. Acta Pol. Pharm..

[74] Šter MP, Gorup EC, Klančič D (2009). Polypharmacy and inappropriate drug prescribing in elderly nursing home residents. Zdr. Vestn..

[75] Stojanović M, Vuković M, Jovanović M, Dimitrijević S, Radenković M (2020). GheOP3S tool and START/STOPP criteria version 2 for screening of potentially inappropriate medications and omissions in nursing home residents. J. Eval. Clin. Pract..

[76] Morris JN, Fries BE, Steel K, Ikegami N, Bernabei R, Carpenter GI, Gilgen R, Hirdes JP, Topinková E (1997). Comprehensive Clinical Assessment in Community Setting: Applicability of the MDS-HC. J. Am. Geriatr. Soc..

[77] Sikora, E. Studies on successful aging and longevity: Polish Centenarian Program. *Acta Biochim. Pol.* **47**, 487–489 (2000). 11051214

[78] Mann E (2012). Potentially inappropriate medication in geriatric patients: The Austrian consensus panel list. Wien. Klin. Wochenschr..

[79] Matanović SM, Vlahovic-Palcevski V (2012). Potentially inappropriate medications in the elderly: A comprehensive protocol. Eur. J. Clin. Pharmacol..

[80] Fialová D, Topinková E, Ballóková A, Matejovska-Kubesova H (2013). 2012 CZ expert consensus for potentially inappropriate medication use in old age: Appropriate choice of drugs and drug dosing in geriatric patients (Section I.), drug-disease interactions in the old age (Section II.). Klin. Farmakol. Farm..

[81] Renom-Guiteras A, Meyer G, Thürmann PA (2015). The EU(7)-PIM list: A list of potentially inappropriate medications for older people consented by experts from seven European countries. Eur. J. Clin. Pharmacol..

[82] Laroche ML, Charmes JP, Merle L (2007). Potentially inappropriate medications in the elderly: A French consensus panel list. Eur. J. Clin. Pharmacol..

[83] Tommelein E (2016). Older patients’ prescriptions screening in the community pharmacy: Development of the Ghent Older People’s Prescriptions community Pharmacy Screening (GheOP3S) tool. J. Public Health (Oxf).

[84] McLeod PJ, Huang AR, Tamblyn RM, Gayton DC (1997). Defining inappropriate practices in prescribing for elderly people: A national consensus panel. CMAJ.

[85] Holt S, Schmiedl S, Thürmann PA (2010). Potentially inappropriate medications in the elderly: The PRISCUS list. Dtsch. Arztebl. Int..

[86] Gorenc, K. Clinical evaluation of pharmacist consultant interventions in community health centre Ljutomer in elderly patients treated with polypharmacy (Master’s thesis). (University of Ljubljana, 2017).

[87] von Elm E (2008). The strengthening the reporting of observational studies in epidemiology (STROBE) statement: Guidelines for reporting observational studies. J. Clin. Epidemiol..

[88] Wells, GA. *et al.* The Newcastle-Ottawa Scale (NOS) for assessing the quality of nonrandomised studies in meta-analyses. https://www.ohri.ca/programs/clinical_epidemiology/oxford.asp (2014).

[89] Lee CS, Liew TM (2017). Inappropriate prescribing among older persons in primary care: Protocol for systematic review and meta-analysis of observational studies. BMJ Open.

[90] Ng BJ, Le Couteur DG, Hilmer SN (2018). Deprescribing benzodiazepines in older patients: Impact of interventions targeting physicians, pharmacists, and patients. Drugs Aging.

[91] Madhusoodanan S, Bogunovic OJ (2004). Safety of benzodiazepines in the geriatric population. Expert Opin. Drug Saf..

[92] Higgins, J. P. T. *et al. Cochrane Handbook for Systematic Reviews of Interventions version 6.1 (updated September 2020)*. (Cochrane, 2021).

