# Author Correction: Prevalence of potentially inappropriate prescribing in older adults in Central and Eastern Europe: a systematic review and synthesis without meta-analysis

**DOI:** 10.1038/s41598-022-25155-9

**Published:** 2022-12-09

**Authors:** Jovana Brkic, Daniela Fialova, Betul Okuyan, Ingrid Kummer, Sofija Sesto, Andreas Capiau, Maja Ortner Hadziabdic, Konstantin Tachkov, Veera Bobrova

**Affiliations:** 1 Department of Social and Clinical Pharmacy, Faculty of Pharmacy in Hradec Kralove, Charles University, Hradec Kralove, 500 05 Czech Republic; 2 Department of Geriatrics and Gerontology, First Faculty of Medicine, Charles University, Prague, 121 08 Czech Republic; 3 Clinical Pharmacy Department, Faculty of Pharmacy, Marmara University, Istanbul, 34668 Turkey; 4 Department of Social Pharmacy and Pharmaceutical Legislation, Faculty of Pharmacy, University of Belgrade, 11221 Belgrade, Serbia; 5 Pharmaceutical Care Unit, Faculty of Pharmaceutical Sciences, Ghent University, 9000 Ghent, Belgium; 6 Department of Pharmacy, Ghent University Hospital, 9000 Ghent, Belgium; 7 Centre for Applied Pharmacy, Faculty of Pharmacy and Biochemistry, University of Zagreb, 10000 Zagreb, Croatia; 8 Department of Organization and Economics of Pharmacy, Faculty of Pharmacy, Medical University of Sofia, Sofia, 1000 Bulgaria; 9 Institute of Pharmacy, Faculty of Medicine, University of Tartu, 50411 Tartu, Estonia

Correction to: *Scientific Reports* 10.1038/s41598-022-19860-8, published online 06 October2022

The Supplementary Information 1 file published with this Article contained an error in the order of references in the Supplementary Information. The original Supplementary Information 1 file is provided below.

This error has now been corrected in the Supplementary Information 1 file that accompanies the original Article.

## Supplementary Information

Supplementary Information.

