# Supplementary material for: Author Correction: Prevalence of potentially inappropriate prescribing in older adults in Central and Eastern Europe: a systematic review and synthesis without meta-analysis
Source: Sci Rep. 2022 Dec 9;12:21304. doi: 10.1038/s41598-022-25155-9 (PMC9734651; doi:10.1038/s41598-022-25155-9)
Supplement: Supplementary file 1 — Supplementary Information. [file 41598_2022_25155_MOESM1_ESM.docx]

**Supplementary Information**

**Supplementary Table S1.** Synthesis without meta-analysis (SWiM) checklist

| **SWiM reporting item** | **Item description** | **Page in manuscript where item is reported** | **Other*** |
| --- | --- | --- | --- |
| *Methods* | | | |
| **1** Grouping studies for synthesis | 1a) Provide a description of, and rationale for, the groups used in the synthesis (e.g., groupings of populations, interventions, outcomes, study design) | Methods, Paragraph 8 |  |
|  | 1b) Detail and provide rationale for any changes made subsequent to the protocol in the groups used in the synthesis | Methods, Paragraph 8 |  |
| **2** Describe the standardised metric and transformation methods used | Describe the standardised metric for each outcome. Explain why the metric(s) was chosen, and describe any methods used to transform the intervention effects, as reported in the study, to the standardised metric, citing any methodological guidance consulted | Methods, Paragraph 7 |  |
| **3** Describe the synthesis methods | Describe and justify the methods used to synthesise the effects for each outcome when it was not possible to undertake a meta-analysis of effect estimates | Methods, Paragraph 9 |  |
| **4** Criteria used to prioritise results for summary and synthesis | Where applicable, provide the criteria used, with supporting justification, to select the particular studies, or a particular study, for the main synthesis or to draw conclusions from the synthesis (e.g., based on study design, risk of bias assessments, directness in relation to the review question) | Methods, Paragraph 8 |  |
| **SWiM reporting item** | **Item description** | **Page in manuscript where item is reported** | **Other*** |
| **5** Investigation of heterogeneity in reported effects | State the method(s) used to examine heterogeneity in reported effects when it was not possible to undertake a meta-analysis of effect estimates and its extensions to investigate heterogeneity | Methods, Paragraph 10 |  |
| **6** Certainty of evidence | Describe the methods used to assess certainty of the synthesis findings | Methods, Paragraph 11 |  |
| **7** Data presentation methods | Describe the graphical and tabular methods used to present the effects (e.g., tables, forest plots, harvest plots).  Specify key study characteristics (e.g., study design, risk of bias) used to order the studies, in the text and any tables or graphs, clearly referencing the studies included | Methods, Paragraphs 5 and 9 |  |
| *Results* | | | |
| **8** Reporting results | For each comparison and outcome, provide a description of the synthesised findings, and the certainty of the findings. Describe the result in language that is consistent with the question the synthesis addresses, and indicate which studies contribute to the synthesis | Results, Paragraph 10, Table 3, and Figure 3a |  |
| *Discussion* |  |  |  |
| **9** Limitations of the synthesis | Report the limitations of the synthesis methods used and/or the groupings used in the synthesis, and how these affect the conclusions that can be drawn in relation to the original review question | Methods, Paragraph 9 |  |

**Supplementary Table S2.** Preferred reporting items for systematic reviews and meta-analyses (PRISMA) checklist

| **Section and Topic** | **Item #** | **Checklist item** | **Location where item is reported** |
| --- | --- | --- | --- |
| **TITLE** | | |  |
| Title | 1 | Identify the report as a systematic review. | Title |
| **ABSTRACT** | | |  |
| Abstract | 2 | See the PRISMA 2020 for Abstracts checklist. | Supplementary Table S3 |
| **INTRODUCTION** | | |  |
| Rationale | 3 | Describe the rationale for the review in the context of existing knowledge. | Introduction, Paragraph 4 |
| Objectives | 4 | Provide an explicit statement of the objective(s) or question(s) the review addresses. | Introduction, Paragraph 5 |
| **METHODS** | | |  |
| Eligibility criteria | 5 | Specify the inclusion and exclusion criteria for the review and how studies were grouped for the syntheses. | Methods, Paragraph 3 |
| Information sources | 6 | Specify all databases, registers, websites, organisations, reference lists and other sources searched or consulted to identify studies. Specify the date when each source was last searched or consulted. | Methods, Paragraph 2, and Supplementary tables S5 and S6 |
| Search strategy | 7 | Present the full search strategies for all databases, registers and websites, including any filters and limits used. | Methods, Paragraph 2, and Supplementary tables S5 and S6 |
| Selection process | 8 | Specify the methods used to decide whether a study met the inclusion criteria of the review, including how many reviewers screened each record and each report retrieved, whether they worked independently, and if applicable, details of automation tools used in the process. | Methods, Paragraphs 1 and 4 |
| Data collection process | 9 | Specify the methods used to collect data from reports, including how many reviewers collected data from each report, whether they worked independently, any processes for obtaining or confirming data from study investigators, and if applicable, details of automation tools used in the process. | Methods, Paragraphs 1 and 5 |
| Data items | 10a | List and define all outcomes for which data were sought. Specify whether all results that were compatible with each outcome domain in each study were sought (e.g. for all measures, time points, analyses), and if not, the methods used to decide which results to collect. | Methods, Paragraph 7 |
|  | 10b | List and define all other variables for which data were sought (e.g. participant and intervention characteristics, funding sources). Describe any assumptions made about any missing or unclear information. | Methods, Paragraphs 1 and 5 |
| Study risk of bias assessment | 11 | Specify the methods used to assess risk of bias in the included studies, including details of the tool(s) used, how many reviewers assessed each study and whether they worked independently, and if applicable, details of automation tools used in the process. | Methods, Paragraphs 1 and 6 |
| Effect measures | 12 | Specify for each outcome the effect measure(s) (e.g. risk ratio, mean difference) used in the synthesis or presentation of results. | Methods, Paragraph 7 |
| Synthesis methods | 13a | Describe the processes used to decide which studies were eligible for each synthesis (e.g. tabulating the study intervention characteristics and comparing against the planned groups for each synthesis (item #5)). | Methods, Paragraph 8 |
|  | 13b | Describe any methods required to prepare the data for presentation or synthesis, such as handling of missing summary statistics, or data conversions. | Methods, Paragraph 7 |
|  | 13c | Describe any methods used to tabulate or visually display results of individual studies and syntheses. | Methods, Paragraph 5 |
|  | 13d | Describe any methods used to synthesize results and provide a rationale for the choice(s). If meta-analysis was performed, describe the model(s), method(s) to identify the presence and extent of statistical heterogeneity, and software package(s) used. | Methods, Paragraph 9 |
|  | 13e | Describe any methods used to explore possible causes of heterogeneity among study results (e.g. subgroup analysis, meta-regression). | Methods, Paragraph 10 |
|  | 13f | Describe any sensitivity analyses conducted to assess robustness of the synthesized results. | Methods, Paragraph 12 |
| Reporting bias assessment | 14 | Describe any methods used to assess risk of bias due to missing results in a synthesis (arising from reporting biases). | Methods, Paragraph 6 |
| Certainty assessment | 15 | Describe any methods used to assess certainty (or confidence) in the body of evidence for an outcome. | Methods, Paragraph 11 |
| **RESULTS** | | |  |
| Study selection | 16a | Describe the results of the search and selection process, from the number of records identified in the search to the number of studies included in the review, ideally using a flow diagram. | Results, Paragraphs 1 and 2, and Figure 1 |
|  | 16b | Cite studies that might appear to meet the inclusion criteria, but which were excluded, and explain why they were excluded. | Supplementary Table S8 |
| Study characteristics | 17 | Cite each included study and present its characteristics. | Results, Paragraphs 3, 4, 5, 6 and 7, and Table 1 |
| Risk of bias in studies | 18 | Present assessments of risk of bias for each included study. | Results, Paragraph 8, Figure 2, Supplementary Figure S1, and Table 2 |
| Results of individual studies | 19 | For all outcomes, present, for each study: (a) summary statistics for each group (where appropriate) and (b) an effect estimate and its precision (e.g. confidence/credible interval), ideally using structured tables or plots. | Results, Paragraph 10, and Table 2 |
| Results of syntheses | 20a | For each synthesis, briefly summarise the characteristics and risk of bias among contributing studies. | Results, Paragraph 10, and Table 2 |
|  | 20b | Present results of all statistical syntheses conducted. If meta-analysis was done, present for each the summary estimate and its precision (e.g. confidence/credible interval) and measures of statistical heterogeneity. If comparing groups, describe the direction of the effect. | Results, Paragraph 10, Table 3, and Figure 3a |
|  | 20c | Present results of all investigations of possible causes of heterogeneity among study results. | Results, Paragraph 12, and Figure 3 |
|  | 20d | Present results of all sensitivity analyses conducted to assess the robustness of the synthesized results. | Results, Paragraph 13, and Supplementary Table S10 |
| Reporting biases | 21 | Present assessments of risk of bias due to missing results (arising from reporting biases) for each synthesis assessed. | Results, Paragraph 9 |
| Certainty of evidence | 22 | Present assessments of certainty (or confidence) in the body of evidence for each outcome assessed. | Results, Paragraph 10, Discussion, Paragraph 7, and Table 3 |
| **DISCUSSION** | | |  |
| Discussion | 23a | Provide a general interpretation of the results in the context of other evidence. | Discussion, Paragraphs 2, 3 and 4 |
|  | 23b | Discuss any limitations of the evidence included in the review. | Discussion, Paragraph 7 |
|  | 23c | Discuss any limitations of the review processes used. | Discussion, Paragraph 9 |
|  | 23d | Discuss implications of the results for practice, policy, and future research. | Conclusions, Paragraphs 2 and 3 |
| **OTHER INFORMATION** | | |  |
| Registration and protocol | 24a | Provide registration information for the review, including register name and registration number, or state that the review was not registered. | Abstract and Methods, Paragraph 1 |
|  | 24b | Indicate where the review protocol can be accessed, or state that a protocol was not prepared. | Abstract and Methods, Paragraph 1 |
|  | 24c | Describe and explain any amendments to information provided at registration or in the protocol. | Supplementary Table S7 |
| Support | 25 | Describe sources of financial or non-financial support for the review, and the role of the funders or sponsors in the review. | Funding |
| Competing interests | 26 | Declare any competing interests of review authors. | Competing interests |
| Availability of data, code and other materials | 27 | Report which of the following are publicly available and where they can be found: template data collection forms; data extracted from included studies; data used for all analyses; analytic code; any other materials used in the review. | Data availability |

**Supplementary Table S3.** The PRISMA for Abstracts Checklist

| **Section and Topic** | **Item #** | **Checklist item** | **Reported (Yes/No)** |
| --- | --- | --- | --- |
| **TITLE** | | |  |
| Title | 1 | Identify the report as a systematic review. | Yes |
| **BACKGROUND** | | |  |
| Objectives | 2 | Provide an explicit statement of the main objective(s) or question(s) the review addresses. | Yes |
| **METHODS** | | |  |
| Eligibility criteria | 3 | Specify the inclusion and exclusion criteria for the review. | Yes |
| Information sources | 4 | Specify the information sources (e.g. databases, registers) used to identify studies and the date when each was last searched. | Yes |
| Risk of bias | 5 | Specify the methods used to assess risk of bias in the included studies. | Yes |
| Synthesis of results | 6 | Specify the methods used to present and synthesise results. | Yes |
| **RESULTS** | | |  |
| Included studies | 7 | Give the total number of included studies and participants and summarise relevant characteristics of studies. | Yes |
| Synthesis of results | 8 | Present results for main outcomes, preferably indicating the number of included studies and participants for each. If meta-analysis was done, report the summary estimate and confidence/credible interval. If comparing groups, indicate the direction of the effect (i.e. which group is favoured). | Yes |
| **DISCUSSION** | | |  |
| Limitations of evidence | 9 | Provide a brief summary of the limitations of the evidence included in the review (e.g. study risk of bias, inconsistency and imprecision). | Yes |
| Interpretation | 10 | Provide a general interpretation of the results and important implications. | Yes |
| **OTHER** | | |  |
| Funding | 11 | Specify the primary source of funding for the review. | No (specified in the article) |
| Registration | 12 | Provide the register name and registration number. | Yes |

**Supplementary Table S4.** PRISMA-S Checklist

| **Section/topic** | **#** | **Checklist item** | **Location(s) Reported** |
| --- | --- | --- | --- |
| **INFORMATION SOURCES AND METHODS** | | | |
| Database name | 1 | Name each individual database searched, stating the platform for each. | Methods, Paragraph 2, and Supplementary tables S5 and S6 |
| Multi-database searching | 2 | If databases were searched simultaneously on a single platform, state the name of the platform, listing all of the databases searched. | Not applicable |
| Study registries | 3 | List any study registries searched. | Not applicable |
| Online resources and browsing | 4 | Describe any online or print source purposefully searched or browsed (e.g., tables of contents, print conference proceedings, web sites), and how this was done. | Not applicable |
| Citation searching | 5 | Indicate whether cited references or citing references were examined, and describe any methods used for locating cited/citing references (e.g., browsing reference lists, using a citation index, setting up email alerts for references citing included studies). | Methods, Paragraph 2 |
| Contacts | 6 | Indicate whether additional studies or data were sought by contacting authors, experts, manufacturers, or others. | Methods, Paragraph 1 |
| Other methods | 7 | Describe any additional information sources or search methods used. | Not applicable |
| **SEARCH STRATEGIES** | | | |
| Full search strategies | 8 | Include the search strategies for each database and information source, copied and pasted exactly as run. | Supplementary tables S5 and S6 |
| Limits and restrictions | 9 | Specify that no limits were used, or describe any limits or restrictions applied to a search (e.g., date or time period, language, study design) and provide justification for their use. | Methods, Paragraph 2 |
| Search filters | 10 | Indicate whether published search filters were used (as originally designed or modified), and if so, cite the filter(s) used. | Methods, Paragraph 2 |
| Prior work | 11 | Indicate when search strategies from other literature reviews were adapted or reused for a substantive part or all of the search, citing the previous review(s). | Methods, Paragraph 2 |
| Updates | 12 | Report the methods used to update the search(es) (e.g., rerunning searches, email alerts). | Methods, Paragraph 2 |
| Dates of searches | 13 | For each search strategy, provide the date when the last search occurred. | Methods, Paragraph 2, and Supplementary tables S5 and S6 |
| **PEER REVIEW** | | | |
| Peer review | 14 | Describe any search peer review process. | Not applicable |
| **MANAGING RECORDS** | | | |
| Total Records | 15 | Document the total number of records identified from each database and other information sources. | Results, Paragraphs 1 and 2, and Figure 1 |
| Deduplication | 16 | Describe the processes and any software used to deduplicate records from multiple database searches and other information sources. | Methods, Paragraph 2 |
|  |  |  |  |

**Supplementary Table S5.** Embase search strategy (Embase.com, 1974 to 14 June 2019)

| Number | Search terms | Results |
| --- | --- | --- |
| #1 | 'inappropriate prescribing'/exp | 4573 |
| #2 | ((inappropriat* or appropriat* or optim* or suboptim* or 'sub optim*' or unnecessary or incorrect* or 'in correct*') near/1 (medicine$ or medicat* or prescrib* or prescription* or drug*)):ti,ab | 20008 |
| #3 | ((over near/1 (prescrib* or prescript*)):ti,ab) or overprescrib*:ti,ab or 'over prescrib*':ti,ab | 2408 |
| #4 | ((under near/1 prescrib*):ti,ab) or underprescrib*:ti,ab or 'under prescrib*':ti,ab | 803 |
| #5 | ((beer* or shan$ or mcleod$) near/3 criter*):ti,ab | 1120 |
| #6 | 'stopp criter*':ti,ab or ‘stopp list$’:ti,ab | 358 |
| #7 | 'start criter*':ti,ab or ‘start list$’:ti,ab | 388 |
| #8 | 'screening tool of older person* prescriptions':ti,ab | 58 |
| #9 | 'screening tool to alert doctors to right treatment':ti,ab | 100 |
| #10 | ('fit for the aged' near/3 (criter* or list$ or instrument or classif*)):ti,ab | 28 |
| #11 | ((forta or rasp or priscus) near/3 (criter* or list$ or instrument)):ti,ab | 125 |
| #12 | 'medication appropriateness index*':ti,ab | 178 |
| #13 | 'assessing care of vulnerable elders':ti,ab or acove:ti,ab | 145 |
| #14 | #1 or #2 or #3 or #4 or #5 or #6 or #7 or #8 or #9 or #10 or #11 or #12 or #13 | 25318 |
| #15 | albania* | 4563 |
| #16 | bosnia* or herzegovina | 15628 |
| #17 | bulgaria* | 77851 |
| #18 | 'central and eastern europe*' | 1818 |
| #19 | croat* | 67747 |
| #20 | czech* | 316603 |
| #21 | estonia* | 18866 |
| #22 | hungar* | 212008 |
| #23 | kosovo | 1786 |
| #24 | latvia* | 9051 |
| #25 | lithuania* | 19497 |
| #26 | macedonia* | 11457 |
| #27 | montenegr* | 9190 |
| #28 | poland or polish or pole* | 605172 |
| #29 | romania* | 111759 |
| #30 | serb* | 88682 |
| #31 | slovak* | 69595 |
| #32 | sloven* | 60126 |
| #33 | yugoslav* | 52655 |
| #34 | #15 or #16 or #17 or #18 or #19 or #20 or #21 or #22 or #23 or #24 or #25 or #26 or #27 or #28 or #29 or #30 or #31 or #32 or #33 | 1572524 |
| #35 | #14 and #34 | 1000 |

**Supplementary Table S6.** MEDLINE search strategy (Ovid, 1946 to 16 June 2019)

| Number | Search terms | Results |
| --- | --- | --- |
| #1 | inappropriate prescribing/ | 2678 |
| #2 | potentially inappropriate medication list/ | 300 |
| #3 | ((inappropriat* or appropriat* or optim* or suboptim* or sub-optim* or unnecessary or incorrect* or in-correct*) adj1 (medicine? or medicat* or prescrib* or prescription* or drug*)).ti,ab. | 12407 |
| #4 | ((over adj1 (prescrib* or prescript*)) or (overprescrib* or over-prescrib*)).ti,ab. | 1519 |
| #5 | ((under adj1 prescrib*) or underprescrib* or under-prescrib*).ti,ab. | 530 |
| #6 | ((beer* or shan? or mcleod?) adj3 criter*).ti,ab. | 612 |
| #7 | ("stopp criter*" or "stopp list?").ti,ab. | 148 |
| #8 | ("start criter*" or "start list?").ti,ab. | 163 |
| #9 | "screening tool of older person's prescriptions".ti,ab. | 85 |
| #10 | "screening tool to alert doctors to right treatment".ti,ab. | 62 |
| #11 | ("fit for the aged" adj3 (criter* or list? or instrument or classif*)).ti,ab. | 17 |
| #12 | ((forta or rasp or priscus) adj3 (criter* or list? or instrument)).ti,ab. | 78 |
| #13 | "medication appropriateness index*".ti,ab. | 117 |
| #14 | ("assessing care of vulnerable elders" or acove).ti,ab. | 91 |
| #15 | or/1-14 | 16039 |
| #16 | albania*.af. | 2221 |
| #17 | (bosnia* or herzegovina).af. | 11415 |
| #18 | bulgaria*.af. | 49199 |
| #19 | "central and eastern europe*".af. | 1564 |
| #20 | croat*.af. | 61554 |
| #21 | czech*.af. | 211672 |
| #22 | estonia*.af. | 10959 |
| #23 | hungar* .af. | 125845 |
| #24 | kosovo.af. | 1364 |
| #25 | latvia*.af. | 4665 |
| #26 | lithuania*.af. | 12781 |
| #27 | macedonia*.af. | 6199 |
| #28 | montenegr*.af. | 5364 |
| #29 | (poland or polish or pole*).af. | 449338 |
| #30 | romania*.af. | 71175 |
| #31 | serb*.af. | 56905 |
| #32 | slovak*.af. | 52705 |
| #33 | sloven*.af. | 31126 |
| #34 | yugoslav*.af. | 14931 |
| #35 | or/16-34 | 1090180 |
| #36 | 15 and 35 | 440 |

**Supplementary Table S7.** Differences between protocol and review

| Differences between protocol and review |
| --- |
| We changed the title of the review.  We changed the authors of the review.  We changed the inclusion criteria – we decided to include studies with adults aged < 60 years if study authors provide separate data for older adults (in the protocol, we stated that separate data must be reported in the study).  We changed the name and definition of the outcome to improve the clarity.  We did not pre-specify several methodological approaches because they were not part of Cochrane methodology when our protocol was published: methods to deal with the multiplicity of outcomes, grouping studies for synthesis, data synthesis method, data presentation methods, and investigation of heterogeneity.  We also did not pre-specify the assessment of non-reporting biases.  We could not assess the effect of three pre-specified potential modifiers. On the other hand, we decided post hoc to examine the effect of two other potential modifiers.  We incorporated the GRADE approach and 'Summary of findings' table.  We did not pre-specify the sensitivity analyses because they were related to the changes we made to the protocol. |

**Supplementary Table S8.** Characteristics of excluded studies

| Study | Reason for exclusion |
| --- | --- |
| Battula (2015) ^93^ | Wrong context (non-CEE country) |
| Bien (2018) ^94^ | Wrong outcome measure |
| Bregnhoj (2009) ^95^ | Wrong context (non-CEE country) |
| Burnett (2009) ^96^ | Wrong context (non-CEE country) |
| Conejos Miquel (2010) ^97^ | Wrong context (non-CEE country) |
| Campins (2017) ^98^ | Wrong context (non-CEE country) |
| Corsonello (2009) ^99^ | Wrong context (non-CEE country) |
| Counter (2018) ^100^ | Wrong context (non-CEE country) |
| Crotty (2004) ^101^ | Wrong context (non-CEE country) |
| Dalleur (2012) ^102^ | Wrong context (non-CEE country) |
| Dalleur (2014) ^103^ | Wrong context (non-CEE country) |
| Fick (2008) ^104^ | Wrong context (non-CEE country) |
| Franchi (2016) ^105^ | Wrong context (non-CEE country) |
| Franse (2018) ^106^ | Wrong outcome measure |
| Freeland (2012) ^107^ | Wrong context (non-CEE country) |
| Gallagher (2008 a) ^108^ | Wrong context (non-CEE country) |
| Gallagher (2008 b) ^109^ | Wrong context (non-CEE country) |
| Gallagher (2011) ^110^ | Wrong context (non-CEE country) |
| Garcia-Gollarte (2012) ^111^ | Wrong context (non-CEE country) |
| Gibert (2018) ^112^ | Wrong context (non-CEE country) |
| Grace (2014) ^113^ | Wrong context (non-CEE country) |
| Gutierrez-Valencia (2017) ^114^ | Wrong context (non-CEE country) |
| Hale (2008) ^115^ | Wrong context (non-CEE country) |
| Hamilton (2011) ^116^ | Wrong context (non-CEE country) |
| Heininger-Rothbucher (2003) ^117^ | Wrong context (non-CEE country) |
| Ilic (2015) ^47^ | Wrong outcome measure |
| Kara (2016) ^118^ | Wrong context (non-CEE country) |
| Karandikar (2013) ^119^ | Wrong context (non-CEE country) |
| Kovacevic (2017) ^120^ | Wrong outcome measure |
| Kovacova (2016) ^121^ | Wrong outcome measure |
| Lesende (2013) ^122^ | Wrong context (non-CEE country) |
| Maio (2006) ^123^ | Wrong context (non-CEE country) |
| Mansur (2009) ^124^ | Wrong context (non-CEE country) |
| Mestres (2015) ^125^ | Wrong context (non-CEE country) |
| Ni Chroinin (2016) ^126^ | Wrong context (non-CEE country) |
| Nixdorff (2008) ^127^ | Wrong context (non-CEE country) |
| Onder (2018) ^128^ | Wrong outcome measure |
| Page II (2006) ^129^ | Wrong context (non-CEE country) |
| Piau (2017) ^130^ | Wrong context (non-CEE country) |
| Price (2017) ^131^ | Wrong context (non-CEE country) |
| Primejdie (2012) ^46^ | Wrong outcome measure |
| Saltvedt (2005) ^132^ | Wrong context (non-CEE country) |
| Schmidt-Mende (2017) ^133^ | Wrong context (non-CEE country) |
| Sennesael (2018) ^134^ | Wrong context (non-CEE country) |
| Shade (2017) ^135^ | Wrong context (non-CEE country) |
| Spinewine (2007) ^136^ | Wrong context (non-CEE country) |
| Spore (1997) ^137^ | Wrong context (non-CEE country) |
| Tamblyn (2003) ^138^ | Wrong context (non-CEE country) |
| Wallace (2017) ^139^ | Wrong context (non-CEE country) |
| Wauters (2016) ^140^ | Wrong context (non-CEE country) |
| Wickop (2016) ^141^ | Wrong context (non-CEE country) |
| Zaveri (2010) ^142^ | Wrong context (non-CEE country) |

**Supplementary Table S9.** Studies awaiting classification

| Study |
| --- |
| Buda (2020) ^143^ |
| Buda (2021) ^144^ |
| Cvetkovic (2019) ^145^ |
| Grina (2020) ^146^ |
| Harasani (2020 a) ^147^ |
| Harasani (2020 b)^148^ |
| Jankyova (2020) ^149^ |
| Stojanovic (2021) ^150^ |

**Supplementary Figure S1.** Risk of bias: reviewers' judgements about each risk of bias item for each included study.


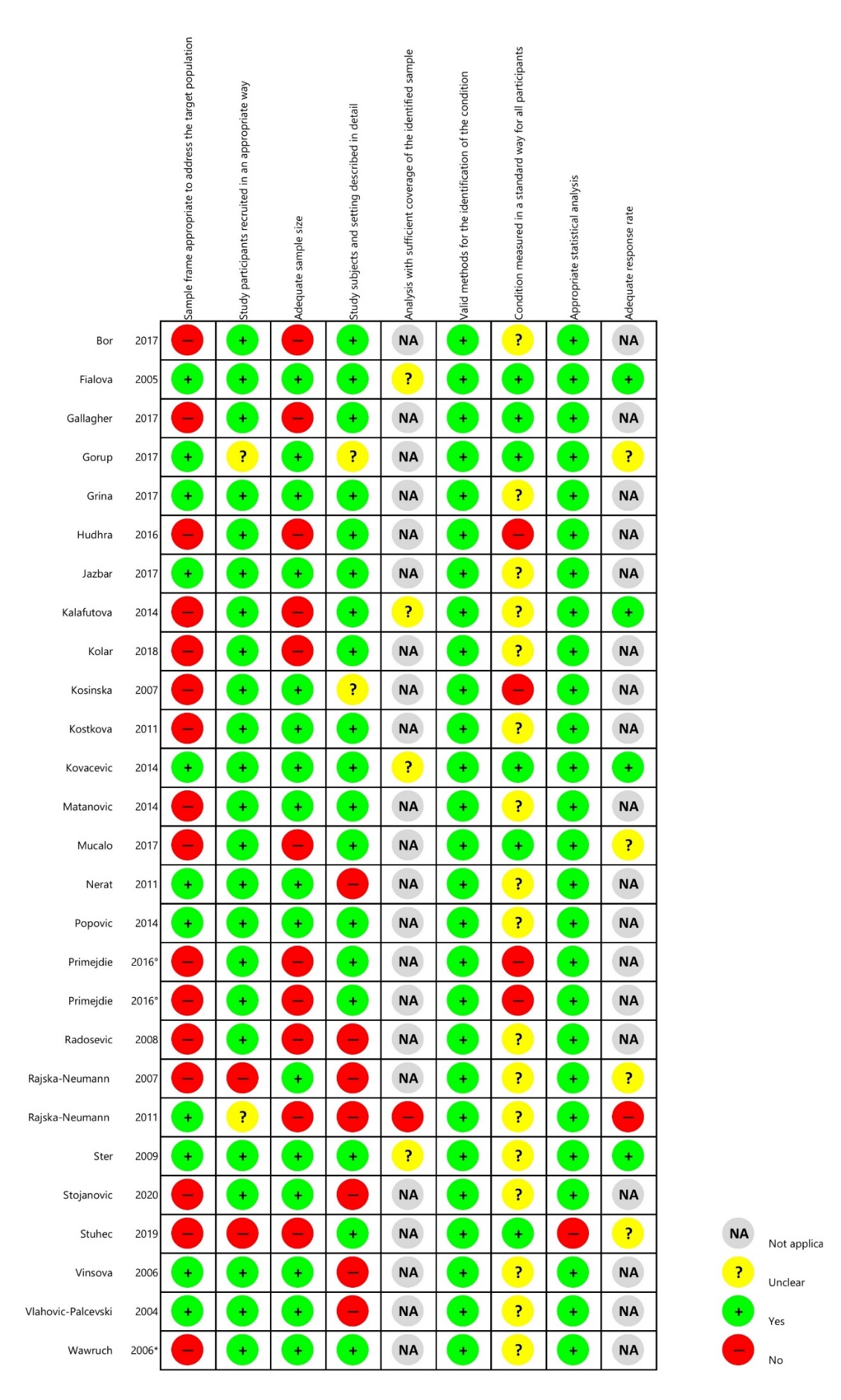


°One report described two studies

∗Indicates the major publication for the study (the study was described in three reports)

**Supplementary Table S10.** Sensitivity analyses

a) Multiplicity of outcomes – using instead of the median outcome, the smallest and the largest outcome for each study

|  | Prevalence median (interquartile range) |  |
| --- | --- | --- |
| Main analysis – Using median outcome | Using smallest outcome | Using largest outcome |
| 34.6 (25.9-63.2) (26 studies) | 34.4 (22.6-56.0) (26 studies) | 52.2 (29.9-63.6) (26 studies) |

b) Eligibility criteria – omitting study with a subset of eligible participants, i.e., Bor et al. ^71^

|  | Prevalence median (interquartile range) | |  |
| --- | --- | --- | --- |
| Main analysis | | Omitting a study with a subset of eligible participants (Bor et al.) ^71^ | |
| 34.6 (25.9-63.2) (26 studies) | | 34.5 (25.8-58.7) (25 studies) | |

**Full list of references**

1. Mekonnen, A. B., Redley, B., de Courten, B. & Manias, E. Potentially inappropriate prescribing and its associations with health-related and system-related outcomes in hospitalised older adults: A systematic review and meta-analysis. *Br. J. Clin. Pharmacol.* **87**, 4150–4172 (2021).

2. Liew, T. M., Lee, C. S., Goh Shawn, K. L. & Chang, Z. Y. Potentially inappropriate prescribing among older persons: A meta-analysis of observational studies. *Ann. Fam. Med.* **17**, 257–266 (2019).

3. Malakouti, S. K. *et al.* A systematic review of potentially inappropriate medications use and related costs among the elderly. *Value Health Reg. Issues* **25**, 172–179 (2021).

4. Cullinan, S., O’Mahony, D., Fleming, A. & Byrne, S. A meta-synthesis of potentially inappropriate prescribing in older patients. *Drugs Aging* **31**, 631–638 (2014).

5. Hill-Taylor, B. *et al.* Effectiveness of the STOPP/START (Screening Tool of Older Persons’ potentially inappropriate Prescriptions/Screening Tool to Alert doctors to the Right Treatment) criteria: Systematic review and meta-analysis of randomized controlled studies. *J. Clin. Pharm. Ther.* **41**, 158–169 (2016).

6. O’Connor, M. N., Gallagher, P. & O’Mahony, D. Inappropriate prescribing: Criteria, detection and prevention. *Drugs Aging* **29**, 437–452 (2012).

7. Beers, M. H. *et al.* Explicit criteria for determining inappropriate medication use in nursing home residents. *Arch. Intern. Med.* **151**, 1825–1832 (1991).

8. Spinewine, A. *et al.* Appropriate prescribing in elderly people: How well can it be measured and optimised?. *Lancet* **370**, 173–184 (2007).

9. Dimitrow, M. S., Airaksinen, M. S. A., Kivelä, S.-L., Lyles, A. & Leikola, S. N. S. Comparison of prescribing criteria to evaluate the appropriateness of drug treatment in individuals aged 65 and older: A systematic review. *J. Am. Geriatr. Soc.* **59**, 1521–1530 (2011).

10. Beers, M. H. Explicit criteria for determining potentially inappropriate medication use by the elderly an update. *Arch. Intern. Med.* **157**, 1531–1536 (1997).

11. Fick, D. M. *et al.* Updating the Beers criteria for potentially inappropriate medication use in older adults: Results of a US consensus panel of experts. *Arch. Intern. Med.* **163**, 2716–2724 (2003).

12. American Geriatrics Society 2012 Beers Criteria Update Expert Panel. American Geriatrics Society updated Beers Criteria for potentially inappropriate medication use in older adults. *J. Am. Geriatr. Soc.* **60**, 616–631 (2012).

13. American Geriatrics Society 2015 Beers Criteria Update Expert Panel. American Geriatrics Society 2015 updated Beers Criteria for potentially inappropriate medication use in older adults. *J. Am. Geriatr. Soc.* **63**, 2227–2246 (2015).

14. 2019 American Geriatrics Society Beers Criteria® Update Expert Panel. American Geriatrics Society 2019 updated AGS Beers Criteria® for potentially inappropriate medication use in older adults. *J. Am. Geriatr. Soc.* **67**, 674–694 (2019).

15. Gallagher, P., Ryan, C., Byrne, S., Kennedy, J. & O’Mahony, D. STOPP (Screening Tool of Older Person’s Prescriptions) and START (Screening Tool to Alert doctors to Right Treatment). Consensus validation. *Int. J. Clin. Pharmacol. Ther.* **46**, 72–83 (2008).

16. O’Mahony, D. *et al.* STOPP/START criteria for potentially inappropriate prescribing in older people: Version 2. *Age Ageing* **44**, 213–218 (2015).

17. Hanlon, J. T. *et al.* A method for assessing drug therapy appropriateness. *J. Clin. Epidemiol.* **45**, 1045–1051 (1992).

18. Clyne, B. *et al.* Interventions to address potentially inappropriate prescribing in community-dwelling older adults: A systematic review of randomized controlled trials. *J. Am. Geriatr. Soc.* **64**, 1210–1222 (2016).

19. Motter, F. R., Fritzen, J. S., Hilmer, S. N., Paniz, É. V. & Paniz, V. M. V. Potentially inappropriate medication in the elderly: A systematic review of validated explicit criteria. *Eur. J. Clin. Pharmacol.* **74**, 679–700 (2018).

20. World Health Organization. Global Health Observatory data repository. Healthy life expectancy (HALE) Data by country. https://apps.who.int/gho/data/view.main.HALEXv?lang=en.

21. World Bank. GDP per capita, PPP (current international $). https://data.worldbank.org/indicator/NY.GDP.PCAP.PP.CD.

22. World Bank. World Bank Country and Lending Groups. https://datahelpdesk.worldbank.org/knowledgebase/articles/906519-world-bank-country-and-lending-groups.

23. Bhagavathula, A. S. *et al.* Prevalence of polypharmacy, hyperpolypharmacy and potentially inappropriate medication use in older adults in India: A systematic review and meta-analysis. *Front. Pharmacol.* **12**, 685518 (2021).

24. Bhagavathula, A. S., Gebreyohannes, E. A. & Fialova, D. Prevalence of polypharmacy and risks of potentially inappropriate medication use in the older population in a developing country: A systematic review and meta-analysis. *Gerontology* **68**, 136–145 (2022).

25. Hill-Taylor, B. *et al.* Application of the STOPP/START criteria: A systematic review of the prevalence of potentially inappropriate prescribing in older adults, and evidence of clinical, humanistic and economic impact. *J. Clin. Pharm. Ther.* **38**, 360–372 (2013).

26. Opondo, D. *et al.* Inappropriateness of medication prescriptions to elderly patients in the primary care setting: A systematic review. *PLoS ONE* **7**, e43617 (2012).

27. Praxedes, M. F. D. S., Pereira, G. C. D. S., Lima, C. F. D. M., Santos, D. B. D. & Berhends J. S. Prescribing potentially inappropriate medications for the elderly according to Beers Criteria: Systematic review. *Cien. Saude Colet.* **26**, 3209–3219 (2021).

28. Thomas, R. E. & Thomas, B. C. A systematic review of studies of the STOPP/START 2015 and American Geriatric Society Beers 2015 criteria in patients ≥ 65 years. *Curr. Aging Sci.* **12**, 121–154 (2019).

29. Storms, H., Marquet, K., Aertgeerts, B. & Claes, N. Prevalence of inappropriate medication use in residential long-term care facilities for the elderly: A systematic review. *Eur. J. Gen. Pract.* **23**, 69–77 (2017).

30. Guaraldo, L., Cano, F. G., Damasceno, G. S. & Rozenfeld, S. Inappropriate medication use among the elderly: A systematic review of administrative databases. *BMC Geriatr.* **11**, 79 (2011).

31. Tommelein, E. *et al.* Potentially inappropriate prescribing in community-dwelling older people across Europe: A systematic literature review. *Eur. J. Clin. Pharmacol.* **71**, 1415–1427 (2015).

32. Liew, T. M., Lee, C. S., Goh, S. K. L. & Chang, Z. Y. The prevalence and impact of potentially inappropriate prescribing among older persons in primary care settings: Multilevel meta-analysis. *Age Ageing* **49**, 570–579 (2020).

33. Morin, L., Laroche, M. L., Texier, G. & Johnell, K. Prevalence of potentially inappropriate medication use in older adults living in nursing homes: A systematic review. *J. Am. Med. Dir. Assoc.* **17**, 862.e1-9 (2016).

34. Brkic, J. *et al.* Prevalence of potentially inappropriate medication use in older adults in Central and Eastern Europe: a systematic review. PROSPERO 2020 CRD42020152713. https://www.crd.york.ac.uk/prospero/display_record.php?ID=CRD42020152713 (2020).

35. Campbell, M. *et al.* Synthesis without meta-analysis (SWiM) in systematic reviews: Reporting guideline. *BMJ* **368**, I6890 (2020).

36. Page, M. J. *et al.* The PRISMA 2020 statement: An updated guideline for reporting systematic reviews. *BMJ* **372**, n71 (2021).

37. Page, M. J. *et al.* PRISMA 2020 explanation and elaboration: Updated guidance and exemplars for reporting systematic reviews. *BMJ* **372**, n160 (2021).

38. Rethlefsen, M. L. *et al.* PRISMA-S: An extension to the PRISMA Statement for Reporting Literature Searches in Systematic Reviews. *Syst. Rev.* **10**, 39 (2021).

39. Alldred, D. P., Kennedy, M. C., Hughes, C., Chen, T. F. & Miller, P. Interventions to optimise prescribing for older people in care homes. *Cochrane Database Syst. Rev.* **2***,* CD009095 (2016).

40. Rankin, A. *et al.* Interventions to improve the appropriate use of polypharmacy for older people. *Cochrane Database Syst. Rev.* **9**, CD008165 (2018).

41. World Health Organization. *Active Ageing: A Policy Framework*. (World Health Organization, 2002).

42. Munn, Z., Moola, S., Lisy, K., Riitano, D. & Tufanaru, C. Methodological guidance for systematic reviews of observational epidemiological studies reporting prevalence and cumulative incidence data. *Int. J. Evid. Based Healthc.* **13**, 147–153 (2015).

43. Guyatt, G. H. *et al.* GRADE: An emerging consensus on rating quality of evidence and strength of recommendations. *BMJ* **336**, 924 (2008).

44. Guyatt, G. *et al.* GRADE guidelines: 1. Introduction—GRADE evidence profiles and summary of findings tables. *J. Clin. Epidemiol.* **64**, 383–394 (2011).

45. GRADE Working Group. Grading quality of evidence and strength of recommendations. *BMJ* **328**, 1490 (2004).

46. Primejdie, D., Bojiţǎ, M. & Popa, A. Potential inappropriate medication use in community - dwelling elderly patients. A qualitative study. *Farmacia* **60**, 366–378 (2012).

47. Ilić, D., Bukumirić, Z. & Janković, S. Impact of educational intervention on prescribing inappropriate medication to elderly nursing homes residents. *Srp. Arh. Celok. Lek.* **143**, 174–179 (2015).

48. Primejdie, D. P., Bojita, M. T. & Popa, A. Potentially inappropriate medications in elderly ambulatory and institutionalized patients: An observational study. *BMC Pharmacol. Toxicol.* **17**, 38 (2016).

49. Wawruch, M. *et al.* Quality indicators of pharmacotherapy in geriatrics. *Klin. Farmakol. Farm.* **20**, 135–139 (2006).

50. Wawruch, M. *et al.* Factors influencing the use of potentially inappropriate medication in older patients in Slovakia. *J. Clin. Pharm. Ther.* **33**, 381–392 (2008).

51. Wawruch, M. *et al.* Perception of potentially inappropriate medication in elderly patients by Slovak physicians. *Pharmacoepidemiol. Drug Saf.* **15**, 829–834 (2006).

52. Stuhec, M., Gorenc, K. & Zelko, E. Evaluation of a collaborative care approach between general practitioners and clinical pharmacists in primary care community settings in elderly patients on polypharmacy in Slovenia: A cohort retrospective study reveals positive evidence for implementation. *BMC Health Serv. Res.* **19**, 118 (2019).

53. Gallagher, P. *et al.* Prevalence of potentially inappropriate prescribing in an acutely ill population of older patients admitted to six European hospitals. *Eur. J. Clin. Pharmacol.* **67**, 1175–1188 (2011).

54. Hudhra, K. *et al.* Prevalence and factors associated with potentially inappropriate prescriptions among older patients at hospital discharge. *J. Eval. Clin. Pract.* **22**, 707–713 (2016).

55. Kostková, L., Mačugová, A., Drobná, V., Dukát, A. & Wawruch, M. Potentially inappropriate prescription in elderly patients: Comparison of selected quality indicators. *Klin. Farmakol. Farm.* **25**, 167–171 (2011).

56. Matanović, S. M. & Vlahović-Palčevski, V. Potentially inappropriate prescribing to the elderly: Comparison of new protocol to Beers criteria with relation to hospitalizations for ADRs. *Eur. J. Clin. Pharmacol.* **70**, 483–490 (2014).

57. Mucalo, I. *et al.* Potentially inappropriate medicines in elderly hospitalised patients according to the EU(7)-PIM list, STOPP version 2 criteria and comprehensive protocol. *Eur. J. Clin. Pharmacol.* **73**, 991–999 (2017).

58. Radošević, N., Gantumur, M. & Vlahović-Palčevski, V. Potentially inappropriate prescribing to hospitalised patients. *Pharmacoepidemiol. Drug Saf.* **17**, 733–737 (2008).

59. Fialová, D. *et al.* Potentially inappropriate medication use among elderly home care patients in Europe. *JAMA* **293**, 1348–1358 (2005).

60. Gorup, E. C. & Šter, M. P. Number of medications or number of diseases: What influences underprescribing?. *Eur. J. Clin. Pharmacol.* **73**, 1673–1679 (2017).

61. Kosińska, K. & Brandys, J. Potentially inappropriate drugs for geriatric patients. *Przegl. Lek.* **64**, 19–23 (2007).

62. Kovačević, S. V. *et al.* Potentially inappropriate prescribing in older primary care patients. *PLoS ONE* **9**, e95536 (2014).

63. Rajska-Neumann, A. & Wieczorowska-Tobis, K. Polypharmacy and potential inappropriateness of pharmaco-logical treatment among community-dwellling elderly patients. *Arch. Gerontol. Geriatr.* **44**, 303–309 (2007).

64. Rajska-Neumann, A. *et al.* Drug consumption among Polish centenarians. *Arch. Gerontol. Geriatr.* **53**, e29–e32 (2011).

65. Grina, D. & Briedis, V. The use of potentially inappropriate medications among the Lithuanian elderly according to Beers and EU(7)-PIM list – a nationwide cross-sectional study on reimbursement claims data. *J. Clin. Pharm. Ther.* **42**, 195–200 (2017).

66. Jazbar, J., Locatelli, I. & Kos, M. Extent and nature of inappropriate medication prescribing among elderly in Slovenia. *Farm. Vestn.* **68**, 145–151 (2017).

67. Nerat, T. & Kos, M. Analysis of inappropriate medication prescribing in Slovenian elderly patients based on the Beers and Laroche criteria. *Zdr. Varst.* **50**, 34–44 (2011).

68. Popović, B. *et al.* Potentially inappropriate prescribing in elderly outpatients in Croatia. *Eur. J. Clin. Pharmacol.* **70**, 737–744 (2014).

69. Vinšová, J. *et al.* Prevalence and longitudinal trends in prescription of potentially inappropriate medications for the elderly in the Czech Republic. *Prakt. Lek.* **86**, 722–728 (2006).

70. Vlahović-Palčevski, V. & Bergman, U. Quality of prescribing for the elderly in Croatia - Computerized pharmacy data can be used to screen for potentially inappropriate prescribing. *Eur. J. Clin. Pharmacol.* **60**, 217–220 (2004).

71. Bor, A. *et al.* Medication use and risk of falls among nursing home residents: A retrospective cohort study. *Int. J. Clin. Pharm.* **39**, 408–415 (2017).

72. Kalafutová, S., Šulcová, H., Jurašková, B. & Vlček, J. A pharmacotherapy of nursing home residents. *Geriatr. Gerontol.* **3**, 65–70 (2014).

73. Kolar, J., Tinkova, B., Ambrus, T. & Tinkova, V. Analysis of pharmacotherapy in senior homes residents. *Acta Pol. Pharm.* **75**, 223–228 (2018).

74. Šter, M. P., Gorup, E. C. & Klančič, D. Polypharmacy and inappropriate drug prescribing in elderly nursing home residents. *Zdr. Vestn.* **78**, 231–240 (2009).

75. Stojanović, M., Vuković, M., Jovanović, M., Dimitrijević, S. & Radenković, M. GheOP3S tool and START/STOPP criteria version 2 for screening of potentially inappropriate medications and omissions in nursing home residents. *J. Eval. Clin. Pract.* **26**, 158–164 (2020).

76. Mann, E. *et al.* Potentially inappropriate medication in geriatric patients: The Austrian consensus panel list. *Wien. Klin. Wochenschr.* **124**, 160–169 (2012).

77. Matanović, S. M. & Vlahovic-Palcevski, V. Potentially inappropriate medications in the elderly: A comprehensive protocol. *Eur. J. Clin. Pharmacol.* **68**, 1123–1138 (2012).

78. Fialová, D., Topinková, E., Ballóková, A. & Matejovska-Kubesova, H. 2012 CZ expert consensus for potentially inappropriate medication use in old age: Appropriate choice of drugs and drug dosing in geriatric patients (Section I.), drug-disease interactions in the old age (Section II.). *Klin. Farmakol. Farm.* **27**, 18–28 (2013).

79. Renom-Guiteras, A., Meyer, G. & Thürmann, P. A. The EU(7)-PIM list: A list of potentially inappropriate medications for older people consented by experts from seven European countries. *Eur. J. Clin. Pharmacol.* **71**, 861–875 (2015).

80. Laroche, M. L., Charmes, J. P. & Merle, L. Potentially inappropriate medications in the elderly: A French consensus panel list. *Eur. J. Clin. Pharmacol.* **63**, 725–731 (2007).

81. Tommelein, E. *et al.* Older patients’ prescriptions screening in the community pharmacy: Development of the Ghent Older People’s Prescriptions community Pharmacy Screening (GheOP3S) tool. *J. Public Health (Oxf)* **38**, e158–e170 (2016).

82. McLeod, P. J., Huang, A. R., Tamblyn, R. M. & Gayton, D. C. Defining inappropriate practices in prescribing for elderly people: A national consensus panel. *CMAJ* **156**, 385–391 (1997).

83. Holt, S., Schmiedl, S. & Thürmann, P. A. Potentially inappropriate medications in the elderly: The PRISCUS list. *Dtsch. Arztebl. Int.* **107**, 543–551 (2010).

84. Morris, J. N. *et al.* Comprehensive clinical assessment in community setting: Applicability of the MDS-HC. *J. Am. Geriatr. Soc.* **45**, 1017–24 (1997).

85. Sikora, E. Studies on successful aging and longevity: Polish Centenarian Program. *Acta Biochim. Pol.* **47**, 487–9 (2000).

86. Gorenc, K. Clinical evaluation of pharmacist consultant interventions in community health centre Ljutomer in elderly patients treated with polypharmacy (Master’s thesis). (University of Ljubljana, 2017).

87. von Elm, E. *et al.* The Strengthening the Reporting of Observational Studies in Epidemiology (STROBE) statement: Guidelines for reporting observational studies. *J. Clin. Epidemiol.* **61**, 344–349 (2008).

88. Wells, GA. *et al.* The Newcastle-Ottawa Scale (NOS) for assessing the quality of nonrandomised studies in meta-analyses. *https://www.ohri.ca/programs/clinical_epidemiology/oxford.asp* (2014).

89. Lee, C. S. & Liew, T. M. Inappropriate prescribing among older persons in primary care: Protocol for systematic review and meta-analysis of observational studies. *BMJ Open* **7**, e015395 (2017).

90. Ng, B. J., Le Couteur, D. G. & Hilmer, S. N. Deprescribing benzodiazepines in older patients: Impact of interventions targeting physicians, pharmacists, and patients. *Drugs Aging* **35**, 493–521 (2018).

91. Madhusoodanan, S. & Bogunovic, O. J. Safety of benzodiazepines in the geriatric population. *Expert Opin. Drug Saf.* **3**, 485–493 (2004).

92. Higgins, J. P. T. *et al.* *Cochrane Handbook for Systematic Reviews of Interventions version 6.1 (updated September 2020)*. (Cochrane, 2021).

93. Battula, P., Shekar, K. C., Mohan, C. K., Prasad, D. T. S. & Ranganayakulu, D. A pilot study on inappropriate drug utilization in geriatric patients at a tertiary care teaching hospital. *J. Glob. Trends Pharm. Sci.* **6**, 2840–2847 (2015).

94. Bień, B. & Bień-Barkowska, K. Prescribing or deprescribing in older persons: What are the real-life concerns in geriatric practice?. *Pol. Arch. Intern. Med.* **128**, 200–208 (2018).

95. Bregnhøj, L., Thirstrup, S., Kristensen, M. B., Bjerrum, L. & Sonne, J. Combined intervention programme reduces inappropriate prescribing in elderly patients exposed to polypharmacy in primary care. *Eur. J. Clin. Pharmacol.* **65**, 199–207 (2009).

96. Burnett, K. M., Scott, M. G., Fleming, G. F., Clark, C. M. & McElnay, J. C. Effects of an integrated medicines management program on medication appropriateness in hospitalized patients. *Am. J. Health. Syst. Pharm.* **66**, 854–859 (2009).

97. Conejos Miquel, M. D. *et al.* Potentially inappropriate drug prescription in older subjects across health care settings. *Eur. Geriatr. Med.* **1**, 9–14 (2010).

98. Campins, L. *et al.* Randomized controlled trial of an intervention to improve drug appropriateness in community-dwelling polymedicated elderly people. *Fam. Pract.* **34**, 36–42 (2017).

99. Corsonello, A. *et al.* Potentially inappropriate medications and functional decline in elderly hospitalized patients. *J. Am. Geriatr. Soc.* **57**, 1007–1014 (2009).

100. Counter, D., Millar, J. W. T. & McLay, J. S. Hospital readmissions, mortality and potentially inappropriate prescribing: A retrospective study of older adults discharged from hospital. *Br. J. Clin. Pharmacol.* **84**, 1757–1763 (2018).

101. Crotty, M., Rowett, D., Spurling, L., Giles, L. C. & Phillips, P. A. Does the addition of a pharmacist transition coordinator improve evidence-based medication management and health outcomes in older adults moving from the hospital to a long-term care facility? Results of a randomized, controlled trial. *Am. J. Geriatr. Pharmacother.* **2**, 257–264 (2004).

102. Dalleur, O. *et al.* Inappropriate prescribing and related hospital admissions in frail older persons according to the STOPP and START criteria. *Drugs Aging* **29**, 829–837 (2012).

103. Dalleur, O. *et al.* Reduction of potentially inappropriate medications using the STOPP criteria in frail older inpatients: A randomised controlled study. *Drugs Aging* **31**, 291–8 (2014).

104. Fick, D. M., Mion, L. C., Beers, M. H. & Waller, J. L. Health outcomes associated with potentially inappropriate medication use in older adults. *Res. Nurs. Health* **31**, 42–51 (2008).

105. Franchi, C. *et al.* E-learning in order to improve drug prescription for hospitalized older patients: A cluster-randomized controlled study. *Br. J. Clin. Pharmacol.* **82**, 53–63 (2016).

106. Franse, C. B. *et al.* The effectiveness of a coordinated preventive care approach for healthy ageing (UHCE) among older persons in five European cities: A pre-post controlled trial. *Int. J. Nurs. Stud.* **88**, 153–162 (2018).

107. Freeland, K. N. *et al.* Medication use and associated risk of falling in a geriatric outpatient population. *Ann. Pharmacother.* **46**, 1188–1192 (2012).

108. Gallagher, P. F., Barry, P. J., Ryan, C., Hartigan, I. & O’Mahony, D. Inappropriate prescribing in an acutely ill population of elderly patients as determined by Beers’ Criteria. *Age Ageing* **37**, 96–101 (2008).

109. Gallagher, P. & O’Mahony, D. STOPP (Screening Tool of Older Persons’ potentially inappropriate Prescriptions): Application to acutely ill elderly patients and comparison with Beers’ criteria. *Age Ageing* **37**, 673–679 (2008).

110. Gallagher, P. F., O’Connor, M. N. & O’Mahony, D. Prevention of potentially inappropriate prescribing for elderly patients: A randomized controlled trial using STOPP/START criteria. *Clin. Pharmacol. Ther.* **89**, 845–854 (2011).

111. García-Gollarte, F., Baleriola-Júlvez, J., Ferrero-López, I. & Cruz-Jentoft, A. J. Inappropriate drug prescription at nursing home admission. *J. Am. Med. Dir. Assoc.* **13**, 83.e9-83.e15 (2012).

112. Gibert, P. *et al.* Optimizing medication use in elderly people in primary care: Impact of STOPP criteria on inappropriate prescriptions. *Arch. Gerontol. Geriatr.* **75**, 16–19 (2018).

113. Grace, A. R. *et al.* A comparison of Beers and STOPP criteria in assessing potentially inappropriate medications in nursing home residents attending the emergency department. *J. Am. Med. Dir. Assoc.* **15**, 830–834 (2014).

114. Gutiérrez-Valencia, M. *et al.* Impact of hospitalization in an acute geriatric unit on polypharmacy and potentially inappropriate prescriptions: A retrospective study. *Geriatr. Gerontol. Int.* **17**, 2354–2360 (2017).

115. Hale, L. D. S. *et al.* Potentially inappropriate medication use in hospitalized older adults: A DUE using the full Beers criteria. *Formulary* **43**, 326–339 (2008).

116. Hamilton, H., Gallagher, P., Ryan, C., Byrne, S. & O’Mahony, D. Potentially inappropriate medications defined by STOPP criteria and the risk of adverse drug events in older hospitalized patients. *Arch. Intern. Med.* **171**, 1013–1019 (2011).

117. Heininger-Rothbucher, D. *et al.* Problematic drugs in elderly patients presenting to a European emergency room. *Eur. J. Intern. Med.* **14**, 372–376 (2003).

118. Kara, Ö. *et al.* Potentially inappropriate prescribing according to the STOPP/START criteria for older adults. *Aging Clin. Exp. Res.* **28**, 761–768 (2016).

119. Karandikar, Y. S., Chaudhari, S. R., Dalal, N. P., Sharma, M. & Pandit, V. A. Inappropriate prescribing in the elderly: A comparison of two validated screening tools. *J. Clin. Gerontol. Geriatr.* **4**, 109–114 (2013).

120. Kovačević, S. V. *et al.* Evaluation of drug-related problems in older polypharmacy primary care patients. *J. Eval. Clin. Pract.* **23**, 860–865 (2017).

121. Kováčová, B. & Ďurišová, A. Drug-related problems identified by pharmacist-led medication review in Slovak hospitalised patients. *Pharmazie* **71**, 548–551 (2016).

122. Lesende, I. M. *et al.* Potentiality of STOPP/START criteria used in primary care to effectively change inappropriate prescribing in elderly patients. *Eur. Geriatr. Med.* **4**, 293–298 (2013).

123. Maio, V. *et al.* Potentially inappropriate prescribing for elderly patients in 2 outpatient settings. *Am. J. Med. Qual.* **21**, 162–168 (2006).

124. Mansur, N., Weiss, A. & Beloosesky, Y. Is there an association between inappropriate prescription drug use and adherence in discharged elderly patients?. *Ann. Pharmacother.* **43**, 177–184 (2009).

125. Mestres, C., Agustí, A., Puerta, L. & Barba, M. Prescription of potentially inappropriate drugs for geriatric patients in long-term care: Improvement through pharmacist’s intervention. *Eur. J. Hosp. Pharm.* **22**, 198–201 (2015).

126. Ní Chróinín, D. *et al.* Potentially inappropriate medications (PIMs) in older hospital in-patients: Prevalence, contribution to hospital admission and documentation of rationale for continuation. *Australas. J. Ageing* **35**, 262–265 (2016).

127. Nixdorff, N. *et al.* Potentially inappropriate medications and adverse drug effects in elders in the ED. *Am. J. Emerg. Med.* **26**, 697–700 (2008).

128. Onder, G. *et al.* Interactions between drugs and geriatric syndromes in nursing home and home care: Results from Shelter and IBenC projects. *Aging Clin. Exp. Res.* **30**, 1015–1021 (2018).

129. Page II, R. L. & Ruscin, J. M. The risk of adverse drug events and hospital-related morbidity and mortality among older adults with potentially inappropriate medication use. *Am. J. Geriatr. Pharmacother.* **4**, 297–305 (2006).

130. Piau, A. *et al.* Optimization of drug therapy in elderly individuals admitted to a geriatric unit. *Clin. Interv. Aging* **12**, 1691–1696 (2017).

131. Price, M. & Davies, I. Applying STOPP guidelines in primary care through electronic medical record decision support: Randomized control trial highlighting the importance of data quality. *JMIR Med. Inform.* **5**, e15 (2017).

132. Saltvedt, I. *et al.* Patterns of drug prescription in a geriatric evaluation and management unit as compared with the general medical wards: A randomised study. *Eur. J. Clin. Pharmacol.* **61**, 921–928 (2005).

133. Schmidt-Mende, K., Andersen, M., Wettermark, B. & Hasselström, J. Educational intervention on medication reviews aiming to reduce acute healthcare consumption in elderly patients with potentially inappropriate medicines—A pragmatic open-label cluster-randomized controlled trial in primary care. *Pharmacoepidemiol. Drug Saf.* **26**, 1347–1356 (2017).

134. Sennesael, A. L. *et al.* Implementing a screening tool to improve prescribing in hospitalized older patients: A pilot study. *Int. J. Clin. Pharm.* **40**, 15–19 (2018).

135. Shade, M. Y. *et al.* Factors associated with potentially inappropriate medication use in rural, community-dwelling older adults. *J. Gerontol. Nurs.* **43**, 21–30 (2017).

136. Spinewine, A. *et al.* Effect of a collaborative approach on the quality of prescribing for geriatric inpatients: A randomized, controlled trial. *J. Am. Geriatr. Soc.* **55**, 658–665 (2007).

137. Spore, D. L., Mor, V., Larrat, P., Hawes, C. & Hiris, J. Inappropriate drug prescriptions for elderly residents of board and care facilities. *Am. J. Public Health* **87**, 404-409 (1997).

138. Tamblyn, R. *et al.* The medical office of the 21st century (MOXXI): Effectiveness of computerized decision-making support in reducing inappropriate prescribing in primary care. *CMAJ* **169**, 549–556 (2003).

139. Wallace, E., McDowell, R., Bennett, K., Fahey, T. & Smith, S. M. Impact of potentially inappropriate prescribing on adverse drug events, health related quality of life and emergency hospital attendance in older people attending general practice: A prospective cohort study. *J. Gerontol. A. Biol. Sci. Med. Sci.* **72**, 271–277 (2017).

140. Wauters, M. *et al.* Too many, too few, or too unsafe?. Impact of inappropriate prescribing on mortality, and hospitalization in a cohort of community-dwelling oldest old. *Br. J. Clin. Pharmacol.* **82**, 1382–1392 (2016).

141. Wickop, B. *et al.* Potentially inappropriate medication use in multimorbid elderly inpatients: Differences between the FORTA, PRISCUS and STOPP ratings. *Drugs Real World Outcomes* **3**, 317–325 (2016).

142. Zaveri, H. G., Mansuri, S. M. & Patel, V. J. Use of potentially inappropriate medicines in elderly: A prospective study in medicine out-patient department of a tertiary care teaching hospital. *Indian J. Pharmacol.* **42**, 95–98 (2010).

143. Buda, V. *et al.* Potentially inappropriate prescriptions in ambulatory elderly patients living in rural areas of Romania using STOPP/START (Version 2) criteria. *Clin. Interv. Aging* **15**, 407–417 (2020).

144. Buda, V. *et al.* Prescription habits related to chronic pathologies of elderly people in primary care in the western part of Romania: Current practices, international recommendations, and future perspectives regarding the overuse and misuse of medicines. *Int. J. Environ. Res. Public. Health* **18**, 7043 (2021).

145. Cvetkovic, Z., Peric, A. & Dobric, S. Potentially inappropriate prescribing and potential clinically significant drug-drug interactions in older outpatients: Is there any association?. *Med. Kaunas Lith.* **55**, 332 (2019).

146. Grina, D., Karpaviciute, J., Minkute, R. & Briedis, V. Impact of hospitalization on potentially inappropriate prescribing: A cross-sectional study in an acute geriatric hospital in Lithuania. *Int. J. Clin. Pharm.* **42**, 903–910 (2020).

147. Harasani, K., Xhafaj, D. & Qipo, O. Prevalence and types of potentially inappropriate prescriptions among older and middle-aged community-dwelling Albanian patients. *Int. J. Risk Saf. Med.* **31**, 5–13 (2020).

148. Harasani, K., Xhafaj, D., Begolli, A. & Olvera-Porcel, M. C. Prevalence of potentially inappropriate prescriptions in primary care and correlates with mild cognitive impairment. *Pharm. Pract.* **18**, 2017 (2020).

149. Jankyova, S., Rubintova, D. & Foltanova, T. The analysis of the use of potentially inappropriate medications in elderly in the Slovak Republic. *Int. J. Clin. Pharm.* **42**, 100–109 (2020).

150. Stojanović, M., Vuković, M., Jovanović, M., Dimitrijević, S. & Radenković, M. Potentially inappropriate medications in Belgrade, Serbia nursing home residents: A Comparison of two approaches. *Eval. Health Prof.* **44**, 180–185 (2021).
